# Indocyanine green colonic perfusion demonstration following robotic da Vinci X inferior mesenteric artery ligation for the treatment of type II endoleak

**DOI:** 10.1002/rcs.2407

**Published:** 2022-04-23

**Authors:** Joel Lambert, Sulaymaan Al Majid, Robert Salaman, Duncan Gavan, Adnan Sheikh, Michael Gill

**Affiliations:** ^1^ Royal Blackburn Hospital East Lancashire Hospitals NHS Trust Blackburn UK; ^2^ Lancaster Medical School Furness College Lancaster University Lancaster UK; ^3^ Bridges Research Group Royal Blackburn Hospitals NHS Trust Blackburn UK; ^4^ University of Central Lancashire Medical School Preston UK

**Keywords:** Da Vinci X, indocyanine green, robotic surgery, vascular endoleak

## Abstract

**Background:**

We describe the technical operative details of the robotic repair of a type II endoleak (T2E) following endovascular abdominal aortic aneurysm repair (EVAR). We demonstrate that indocyanine green (ICG) can be used intra‐operatively to demonstrate perfusion of the colon following ligation of the inferior mesenteric artery (IMA) vessel feeding the aneurysm sac.

**Methods:**

A 74‐year old male underwent EVAR for a 5.8 cm infra‐renal abdominal aortic aneurysm using an E‐Tegra, Jotec Device (JOTEC Gmb, Lotzenäcker 23,D‐72379 Hechingen). Surveillance contrast CT (CTA) over the ensuing 30 months confirmed progressive sac expansion.

**Results:**

ICG confirmed colonic perfusion via the marginals after IMA ligation. Total operative time 56 min < 50 mls blood loss and 1‐day hospital stay. 3‐month follow‐up: CTA and ultrasound demonstrated complete resolution of T2E and adequately perfused colon.

**Conclusion:**

A total robotic approach can be performed safely with intra‐operative ICG used to demonstrate colonic perfusion as an added safety measure.

## 1 INTRODUCTION

Meta‐analysis data^1^ has quoted the pooled prevalence of type II endoleak (T2E) at 22%, with a patent inferior mesenteric artery (IMA) and an increasing number of lumbar vessels being statistically significant predictors of risk for this complication. There is conflicting evidence on how this complication should be managed. Some studies suggest conservative management as a safe approach.^2^ While others suggest a management strategy involving endovascular techniques such as selective embolization via a transarterial, translumbar or transcaval approach^3^ with the option of minimally invasive surgical intervention as a more definitive option. Guidelines have advocated treatment when the sac diameter reaches or exceeds 10 mm.^4^ A handful or case reports and consecutive patient series have reported satisfactory surgical management via a laparoscopic approach.^5^, ^6^, ^7^ While this is safe and feasible, a robotic approach may allow for a better clarity of dissection especially within the context of anatomically disrupted surgical dissection planes. Morelli et al, have demonstrated safe total robotic ligation of the IMA and feeding lumbar vessels in the management of persistent T2E.^8^ The use of Indocyanine green (ICG) in dynamic visualisation of perfusion and function has been established in a range of surgical specialities.^9^ Porta et al. demonstrated its usefulness in the laparoscopic treatment of a T2E.^5^ We hereby further demonstrate the feasibility and safety of a total robotic approach with the added reassurance of ICG perfusion intra‐operatively.

## 2 METHODS

A 74 year old male underwent endovascular abdominal aortic aneurysm repair in 2016 for a 5.8 cm infra‐renal abdominal aortic aneurysm using an E‐Tegra, Jotec Device (JOTEC Gmb). Surveillance CTs over the ensuing 30 months confirmed progressive sac expansion from 5.8 to 7.0 cm. Initial attempts at selective embolization of the (IMA) were unsuccessful. The graft was then accessed under ultrasound scan (USS) guidance and injected with 5 ml Ethylene‐vinyl alcohol copolymer (Onyx) with intra‐procedural resolution of the endoleak. 1‐month follow‐up ultrasound demonstrated a persistent endoleak arising from the IMA. After multi‐disciplinary team (MDT) discussions involving the colorectal team a decision was made on a full robotic approach.

All available CTA and USS imaging over the preceding 24 months were reviewed by the MDT. Failure of previous endovascular techniques was confirmed and persistent T2E secondary to retrograde IMA filling was identified on contrast enhanced ultrasound (CEUS) 4 weeks prior to surgical intervention. All medical and intra‐operative details were recorded. The Da Vinci X robotic system by Intuitive Surgical Inc (Sunnyvale) was used to perform the procedure in its entirety. Patient consent was gained pre‐operatively for video recording and image capture. The patient was positioned in the Lloyd Davis position with right lateral tilt, 15° tilt in the recumbent position and ports as per standard left sided colonic or rectal resection. 5 mm visiport insertion in the left upper quadrant to create pneumoperitoneum before converting to 3 × 8 mm robotic ports and one 12 mm robotic port.

## 3 RESULTS

**FIGURE 1:**
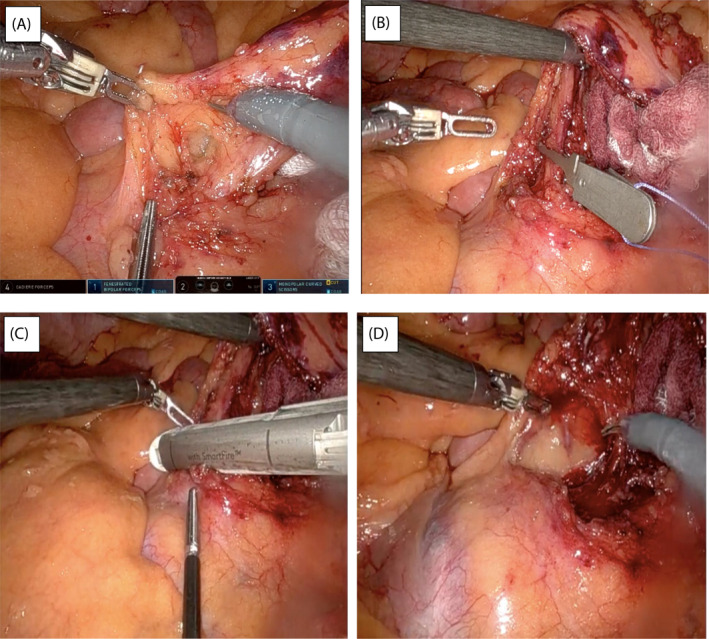
(A) dissection of inferior mesenteric artery (IMA). (B)Application of bulldog clip to check perfusion. (C) Selective ligation of IMA using Smartfire stapling device. (D) Post‐ligation of IMA, aneurysm sac visible in the bottom of image

**FIGURE 2:**
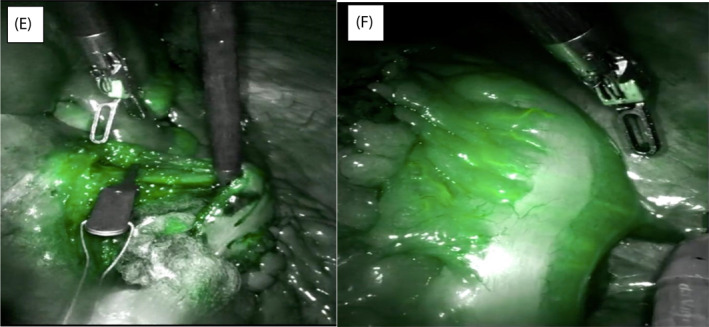
(E) Indocyanine green (ICG) perfusion after bulldog clip applied. (F) perfusion of left colon

**FIGURE 3:**
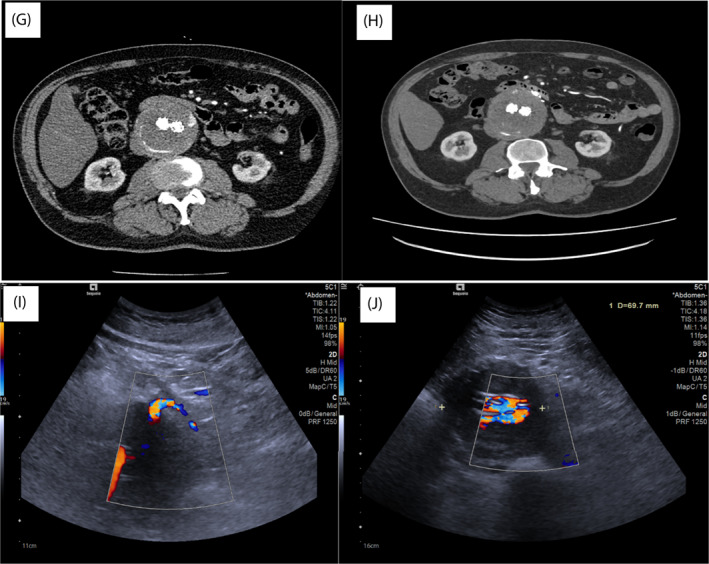
(G) Preoperative CTA with persistent T2E. (H) Postoperative CTA with type II endoleak (T2E) resolved. (I) Preoperative CEUS with inferior mesenteric artery (IMA) filling of sac. (J) Postoperative CEUS with resolved IMA filling

The IMA was identified with minimal adhesions or scarring from previous endovascular therapy and isolated at its origin. Indocyanine green was then used to ensure there were no other collateral vessels adjacent to its root. The left ureter was seen and protected. Baseline perfusion of the left colon was checked with ICG before applying a bulldog clip (Figure 1B) and then re‐checked with a further dose of 2.5 mg ICG (Figure 2E). 45 mm Smartfire robotic stapler was used to divide the IMA then further dose of ICG was used post procedure to ensure continued colonic perfusion (Figure 2F). In total 7.5 mg or the equivalent of 3 ml (2.5 mg in every ml) ICG was given for the entire procedure. ICG given pre and post IMA ligation demonstrated adequate left colonic perfusion via the marginal vessels. Total operative time 56 min with <50 mls blood loss and 1‐day hospital stay. CEUS 4 weeks post operatively demonstrated complete resolution of the T2E (Figure 3J). This was again confirmed on the follow‐up CTA at 3 months (Figure 3H).

## 4 DISCUSSION and CONCLUSION

The main aim of this report was to describe the use of ICG in demonstrating acceptable endoleak stabilisation and colonic perfusion after IMA ligation. This technique of IMA ligation has been previously described by Lin et al,^10^ who did not employ the use of ICG and used a different robotic platform to the one described here.

Robotic surgical systems have afforded the surgeon unique three‐dimensional views and clarity of the surgical field, allowing for more precise dissection especially in anatomically disrupted fields. The use of multiple articulating operative arms allows for complex multitask dissection by a single operator. This innovation can be safely employed across surgical disciplines, with the benefits of reduced length of hospital stay (in our case 24 h) and equivalent operating times to other modalities (such as laparoscopy) with the added benefit of more precise dissection and minimal blood loss.

Guidelines suggest intervention for T2E when the sac diameter exceeds 10 mm due to sustained pressurisation of the sac with increased risk of rupture,^4^ although there is ongoing debate on the precise threshold for intervention.^11^ Best practice guidelines suggest a management algorithm beginning with endovascular techniques involving trans‐arterial approaches followed by operative management.^12^ Multiple series have quoted high failure rates with embolization raising the question of whether definitive surgical management should be considered as first line in selected patients. Our experience confirms inclusion of general surgical input within the vascular MDT may assist in the decision‐making process, provided that such expertise are locally available.

Laparoscopic IMA ligation has been previously described for T2E. While feasible and safe, this approach relies on highly skilled laparoscopic surgeons due to a surgical approach that may be limited by inadequate two‐dimensional views and reduced versatility of function in previously disrupted surgical planes. The fully robotic approach ameliorates these technical issues. We have demonstrated this with the Da Vinci X system introduced by Intuitive Surgical in 2017. In this particular case the main technical challenge was gaining access to the IMA in an anatomical field distorted due to the aneurysm sac (clearly visible in Figure 1D). This would have been challenging to achieve laparoscopically due to and inability to articulate operating instruments at acute angles to allow for safe and precise dissection.

The use of ICG in the dynamic visualisation of organ perfusion has been well established.^9^ Preoperative MDT discussions with the vascular and colorectal teams involved planning for an anterior resection in the event of poor or unsustained perfusion of the colon. The patient was pre‐operatively consented for this eventuality. We have demonstrated that ICG can be used as an intra‐operative adjunct for added reassurance where colonic vascular anatomy may be varied. We suggest that patients with T2E who meet the criteria for surgical intervention, early MDT discussions with the colorectal team may be warranted. If the appropriate expertise and robotic platform is available, this presents a safe and effective option. Considering the high failure rates of endovascular options, there may be an argument to consider surgical intervention as a first line management strategy.

In this case report we have demonstrated that a total robotic approach for the management of T2E can be performed safely with intra‐operative ICG used to demonstrate colonic perfusion as an added safety measure.

## CONFLICT OF INTEREST

For all the authors, the submitted work was not carried out in the presence of any personal, professional or financial relationships that could potentially be construed as a conflict of interest.

## AUTHORS CONTRIBUTION

Joel Lambert wrote the manuscript. Sulaymaan Al Majid, Michael Gill, Adnan Sheikh, Robert Salaman edited the manuscript and verified technical details. Duncan Gavan provided annotated images. All authors have reviewed and approved the manuscript.

## CONSENT FOR PUBLICATION

Written patient consent was gained for video recording and image capture for publication.

## GUIDELINES

CARE guidelines have been adhered to in the production of this manuscript.

## Supporting information

Supporting Information S1

Supporting Information S2

## Data Availability

Data sharing not applicable to this article as no datasets were generated or analysed during the current study.

## References

[1] Guo Q , Du X , Zhao J , et al. Prevalence and risk factors of type II endoleaks after endovascular aneurysm repair: a meta‐analysis. PLoS One. 2017;12:e0170600. 28182753 10.1371/journal.pone.0170600 PMC5300210

[2] Sidloff DA , Gokani V , Stather PW , Choke E , Bown MJ , Sayers RD . Editor’s choice – type II endoleak: conservative management is a safe strategy. Eur J Vasc Endovasc Surg. 2014;48:391‐399. 25042332 10.1016/j.ejvs.2014.06.035

[3] Baum RA , Carpenter JP , Golden MA , et al. Treatment of type 2 endoleaks after endovascular repair of abdominal aortic aneurysms: comparison of transarterial and translumbar techniques. J Vasc Surg. 2002;35:23‐29. 11802129 10.1067/mva.2002.121068

[4] Moll FL , Powell JT , Fraedrich G , et al. Management of abdominal aortic aneurysms clinical practice guidelines of the European society for vascular surgery. Eur J Vasc Endovasc Surg. 2011;41:S1‐S58. 21215940 10.1016/j.ejvs.2010.09.011

[5] Porta M , Cova M , Segreti S , et al. Laparoscopic clipping of the inferior mesenteric artery and intraoperative indocyanine green angiography for type II endoleak following endovascular aneurysm repair. J Laparoendosc Adv Surg Tech. 2020;30:413‐415. 10.1089/lap.2019.0766 31990613

[6] Feezor RJ , Nelson PR , Lee WA , Zingarelli W , Cendan JC . Laparoscopic repair of a type II endoleak. J Laparoendosc Adv Surg Tech. 2006;16:267‐270. 10.1089/lap.2006.16.267 16796438

[7] Ho P , Law WL , Tung PHM , Poon JTC , Ting ACW , Cheng SWK . Laparoscopic transperitoneal clipping of the inferior mesenteric artery for the management of type II endoleak after endovascular repair of an aneurysm. Surg Endosc. 2004;18. 10.1007/s00464-003-4258-1 15216873

[8] Morelli L , Guadagni S , Di Franco G , et al. Technical details and preliminary results of a full robotic type II endoleak treatment with the da Vinci Xi. J Robot Surg. 2019;13:505‐509. 30830571 10.1007/s11701-019-00944-z

[9] Baiocchi GL , Diana M , Boni L . Indocyanine green‐based fluorescence imaging in visceral and hepatobiliary and pancreatic surgery: state of the art and future directions. World J Gastroenterol. 2018;24:2921‐2930. 10.3748/wjg.v24.i27.2921 30038461 PMC6054946

[10] Lin JC , Eun D , Shrivastava A , et al. Total robotic ligation of inferior mesenteric artery for type II endoleak after endovascular aneurysm repair. Ann Vasc Surg. 2009;23:255.e19‐255.e21. 10.1016/j.avsg.2008.02.019 18411030

[11] Karthikesalingam A , Thrumurthy SG , Jackson D , et al. Current evidence is insufficient to define an optimal threshold for intervention in isolated type II endoleak after endovascular aneurysm repair. J Endovasc Ther. 2012;19:200‐208. 10.1583/11-3762R.1 22545885

[12] Bryce Y , Schiro B , Cooper K , et al. Type II endoleaks: diagnosis and treatment algorithm. Cardiovasc Diagn Ther. 2018;8:S131‐S137. 10.21037/cdt.2017.08.06 29850425 PMC5949582

