# Supplementary material for: Indocyanine green colonic perfusion demonstration following robotic da Vinci X inferior mesenteric artery ligation for the treatment of type II endoleak
Source: Int J Med Robot. 2022 Apr 23;18(4):e2407. doi: 10.1002/rcs.2407 (PMC9541556; doi:10.1002/rcs.2407)
Supplement: Supplementary file 1 — Supporting Information S1 [file RCS-18-e2407-s002.pdf]

The Encyclopedia of Medical Robotics Downloaded from www.worldscientific.com
by 5.61.122.207 on 03/25/22. Re-use and distribution is strictly not permitted, except for Open Access articles.

Chapter 1

THE *DA VINCI* SURGICAL SYSTEM

Mahdi Azizian, May Liu, Iman Khalaji, and Simon DiMaio

Intuitive Surgical, Inc. 1020 Kifer Road, Sunnyvale, CA, USA

The *da Vinci*® Surgical System is a platform for robot-assisted minimally invasive surgery (RAMIS). This chapter provides an overview of the design of the *da Vinci* System, as well as several of its key subsystems for vision, tissue manipulation, anatomical access, and operator technology training. Clinical adoption of the system to date is described briefly, and we leave the reader with some thoughts on possible future development directions.

1. Introduction

In this chapter, we describe the *da Vinci*® Surgical System — a platform for robot-assisted minimally invasive surgery (RAMIS). At the time of this writing, there are over 4,400 *da Vinci* Systems installed in more than 60 countries worldwide, with approximately 875,000 procedures performed in 2017. Over 5 million procedures have been completed by surgeons using *da Vinci* Surgical Systems. Despite these numbers, the field of robot-assisted surgery is still relatively early in its evolution. Many exciting technologies are poised to impact the practice of surgery in positive ways and robot-assisted platforms — such as *da Vinci* — will surely play an important role. While this chapter focuses primarily on the *da Vinci* System, we hope that it provides some broader insight into the unique process of invention and development that has resulted in a marriage of technology and medicine that impacts thousands of patients each day.

Take a moment to consider the fundamental goals, or ideals, of surgery. These might include the following abilities: to see diseases perfectly, to eliminate all diseased tissues while sparing healthy tissues, to reconstruct with precision, and to leave the body as if no surgical intervention was ever performed. There are

NOTICE: Prior to using any material contained in any chapter, users are advised to consult with the individual chapter author(s) regarding the material contained in that individual chapter, including but not limited to, their specific design(s) and recommendation(s).

The Encyclopedia of Medical Robotics Downloaded from www.worldscientific.com
by 5.61.122.207 on 03/25/22. Re-use and distribution is strictly not permitted, except for Open Access articles.

several obstacles to achieving these ideals. Perhaps, one of the greatest of these obstacles is that diseased or damaged tissue is rarely easily accessible close to the skin surface. Rather, it is typically buried deep within the structures of the body, such that reaching it can lead to significant collateral damage as the surgeon passes through healthy tissues to reach their target. This has been a fundamental driver for minimally invasive surgical techniques, including laparoscopic surgery.

We can think of laparoscopic surgery as a form of remote visualization and manipulation that uses mechanical instrumentation to transmit the surgeon's motions from the outside of the patient's body to the surgical field inside the patient's body, as well as to capture and return video images from the field, via a smaller set of incisions than might be required for an open surgery. The *da Vinci* system takes this approach a step further by means of teleoperation technologies that place a sophisticated computerized control system between the surgeon and the surgical field, thereby enabling complex tissue manipulations while minimizing invasiveness. The purpose of this is to make the visualization and manipulation of tissue structures as transparent and as natural as possible for the surgeon. This form of teleoperation creates opportunities for going beyond transparency to significantly enhancing the surgeon's natural capabilities by

- Stabilizing motions and reducing physiological tremor,
- Scaling motions to assist with precision tasks,
- Augmenting the surgeon's senses for greater awareness,
- Providing navigation assistance within the anatomy,
- Anticipating and warning the user of critical events, and
- Perhaps one day to automate surgical tasks or subtasks.

Present day technologies barely scratch the surface of these opportunities. The remainder of this chapter describes the *da Vinci* Surgical System today — its basic architecture, key design features, and its product ecosystem, as well as themes for future innovation. Section 2 briefly outlines the history of Intuitive Surgical, the creator of *da Vinci* Systems, and a company timeline between 1995 and 2017. Section 3 describes the basic anatomy of the *da Vinci* Surgical System, as well as some key areas in which this platform has evolved from one generation to the next. Sections 4–6 describe examples of visualization, tissue manipulation, and surgical access aspects that are important to this platform and for which there are interesting opportunities for further innovation. Section 7 describes training challenges and opportunities, Section 8 outlines clinical adoption to date, and finally, Section 9 provides concluding thoughts and future directions.

2. The Intuitive Surgical Timeline

Intuitive Surgical was founded in 1995 by Dr. Frederick Moll, M.D., Rob Younge, and John Freund. The company licensed telepresence surgical technology from SRI (Menlo Park, CA) and began developing what would ultimately become the *da Vinci* Surgical System, the first of which was installed in late 1998. Early marketing and sales of the system were focused outside of the United States, until the company received U.S. Food and Drug Administration (FDA) clearance for the *da Vinci* System in mid-2000. This first FDA clearance was for applications in general surgery; however, additional indications for thoracoscopic (chest) and radical prostatectomy procedures followed one year later.

During this early period, Intuitive Surgical competed with a company called Computer Motion, Inc. — makers of the Zeus® Surgical System that had launched in 1997. The Zeus system was based on an early product called AESOP®, a voice controlled endoscope manipulator that was the first surgical robotic device to receive FDA approval. Initially, the Zeus® was preferred by general laparoscopic surgeons, while the *da Vinci*® was adopted by open surgeons who did not perform laparoscopic surgery. Zeus® was smaller, had a lower price point, but was less capable.

By 1999, Computer Motion began to adjust their approach and started moving toward the course that Intuitive Surgical was on. Competition led to the merger between Intuitive Surgical and Computer Motion in 2003. At that time, the Zeus® system was phased out in favor of the *da Vinci* System, because of the additional capability. Some of these key events of Intuitive Surgical's early history are indicated in a company timeline in Fig. 1. Further details of this period are provided in Ref. [1].

In its second decade, Intuitive Surgical developed and launched a series of products to extend and evolve the *da Vinci* platform. New clinical indications were added as the technology was refined and as surgeons of several different specialties began to adopt system. Five models of the system have been launched globally to date, as illustrated in Fig. 2.[a]

The following section describes basic principles of operation and key design refinements in recent embodiments of the system. Subsequent sections elaborate on the platform subsystems and how these take advantage of the computerized control system that is central to the architecture of *da Vinci* Systems.

[a]As of June 2017, not all products may be available in all markets pending individual country approvals.

The Encyclopedia of Medical Robotics Downloaded from www.worldscientific.com
by 5.61.122.207 on 03/25/22. Re-use and distribution is strictly not permitted, except for Open Access articles.

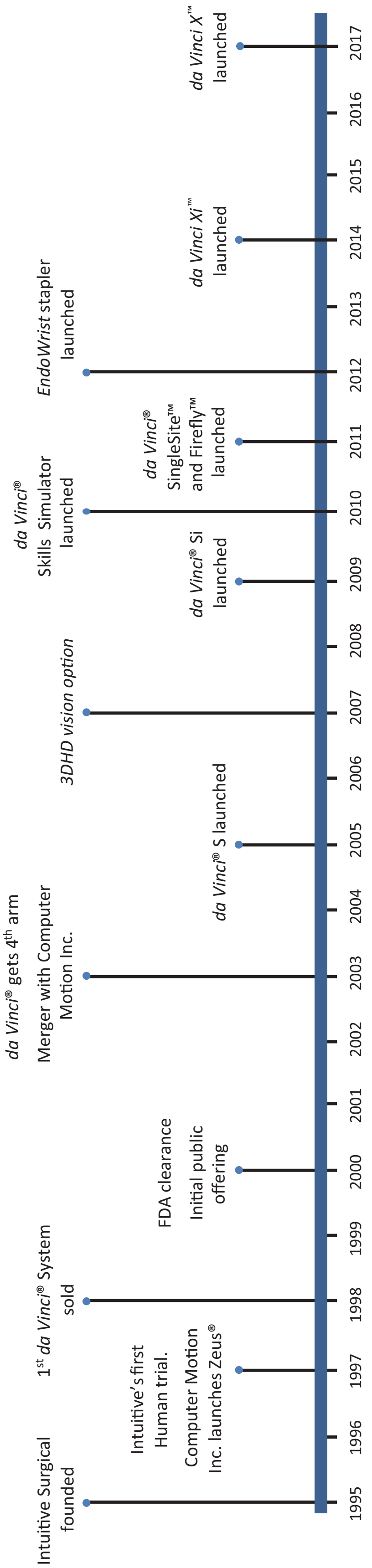

Figure 1. Timeline of selected company milestones.

The Encyclopedia of Medical Robotics Downloaded from www.worldscientific.com by 5.61.122.207 on 03/25/22. Re-use and distribution is strictly not permitted, except for Open Access articles.

The Encyclopedia of Medical Robotics Downloaded from www.worldscientific.com
by 5.61.122.207 on 03/25/22. Re-use and distribution is strictly not permitted, except for Open Access articles.

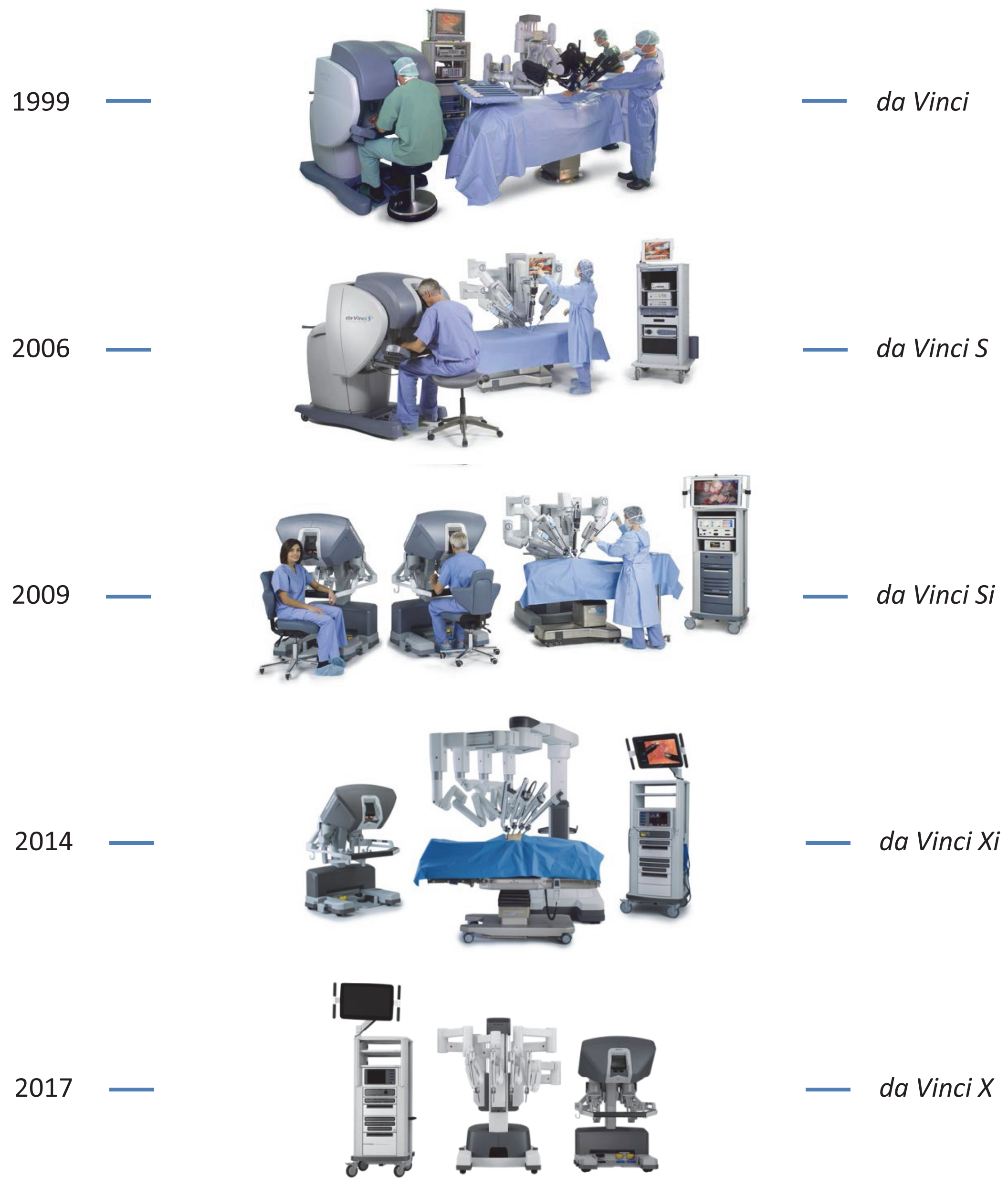

Figure 2. Five models of the *da Vinci* Surgical System (© 2017 Intuitive Surgical, Inc.).

3. Basic Principles and Design of the *da Vinci* Surgical System

The *da Vinci* System comprises three distinct subsystems: (i) the patient-side cart; (ii) the surgeon console; (iii) the vision cart. These subsystems are present in each of the four generations of *da Vinci* Systems that have been marketed to date, as shown in Fig. 2. In this figure, the surgeon is seated at a surgeon console, from which they control the motion of surgical instruments that are situated at the patient side, as well as observe video images from inside the patient. Unlike the laparoscopic approach — which connects the surgeon to the surgical field via a mechanical linkage — the *da Vinci* operates using the principle of teleoperation.

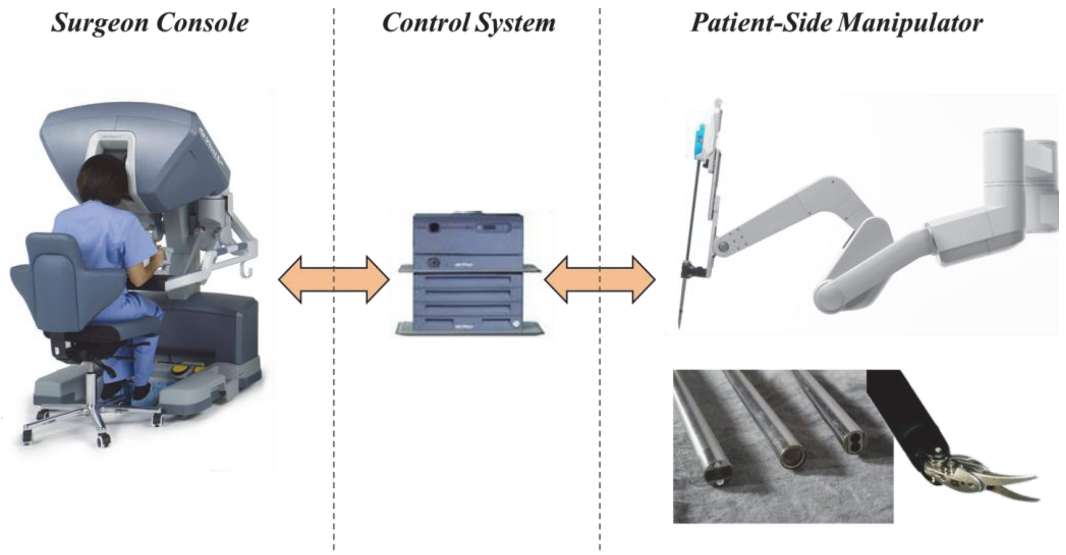

Figure 3. At the heart of the *da Vinci* System is a master–slave teleoperation architecture, with the surgeon console containing two master interfaces that are used to control slave manipulators that are part of the patient side cart (© 2017 Intuitive Surgical, Inc.).

The Encyclopedia of Medical Robotics Downloaded from www.worldscientific.com by 5.61.122.207 on 03/25/22. Re-use and distribution is strictly not permitted, except for Open Access articles.

The idea of "teleoperation" or "telemanipulation" has been contemplated in science fiction writing since the 1940s[2] and has since been deployed to space exploration, deep sea exploration, hazardous material handling, ordinance disposal, and a variety of other applications. A brief history of the evolution of telerobotics in surgery is provided in Ref. [1]. In the context of the *da Vinci* System, this approach relies on an electronic connection between the surgeon's "master interface" and the surgical instruments that are driven by "slave manipulators". A computerized control system acts as an intermediary in this master–slave architecture, and is a key component of the system. The master–slave architecture of the *da Vinci* System is illustrated in Fig. 3.

The patient-side manipulators are mounted to the patient-side cart via a setup structure, which is discussed in detail later. Each manipulator may support either a stereo endoscopic camera or a surgical instrument, such as a grasper, a scissor, or a needle driver. Due to their contact with the patient, these instruments work within the sterile field, whereas the surgeon and the surgeon console remain outside of the sterile field. The computerized control system extends the surgeon's "presence" — their sensory awareness and control — into the surgical field by transmitting video images from the endoscopic camera to the stereo viewer of the console, and transmitting the surgeon's hand motions — measured by the master interfaces — to the slave manipulators. Since this is an electronic link, the software of the control system can modify the signals, so as to filter out the surgeon's normal physiological tremor, or to scale down their motions for enhanced precision.

The control system may also augment the surgeon's view of the anatomy to provide them with navigation and guidance information; or in the future, it may help the surgeon to better anticipate critical task steps. This ability to enhance the

surgeon's capabilities is a key advantage of this type of system and is an aspect that is explored later in this chapter.

When Intuitive Surgical began its development work in late 1995, the product vision included four key specifications ("product pillars"). First and foremost, the system had to be reliable and failsafe in order to be feasible as a surgical device; second, the system was to provide the user with intuitive control of the instrument; third, the instrument tips were to have six-degree-of-freedom (6-DoF) dexterity as well as a functional gripper. The fourth pillar was to provide the surgeon with compelling 3D visualization of the anatomy. By transposing the surgeon's eyes and hands into the patient in a reliable and effective way, these product pillars supported the ultimate goal to provide the surgeon with several key benefits of open surgery that had been lost in the laparoscopic approach, while maintaining minimal invasiveness (Fig. 4).

Subsequent generations of *da Vinci* System have extended these original product pillars to improve ease of use for the patient care team. A coordinated team is needed in order to perform surgery and several members of this team will interact with the *da Vinci* System and its components during the multiple phases of a surgery, which include preparing the system for use, draping the robotic arms, roll-up (positioning the patient cart next to the patient bed), deployment (adjusting the angles of the robotic arms to ensure clearance between the arms and the patient), docking (securing the connection between the robotic arms and the patient), removing and inserting instruments during the operation, undocking, undraping and cleaning, stowing the robotic arms to minimize space required for storage, and reprocessing of instruments and accessories. Examples of key

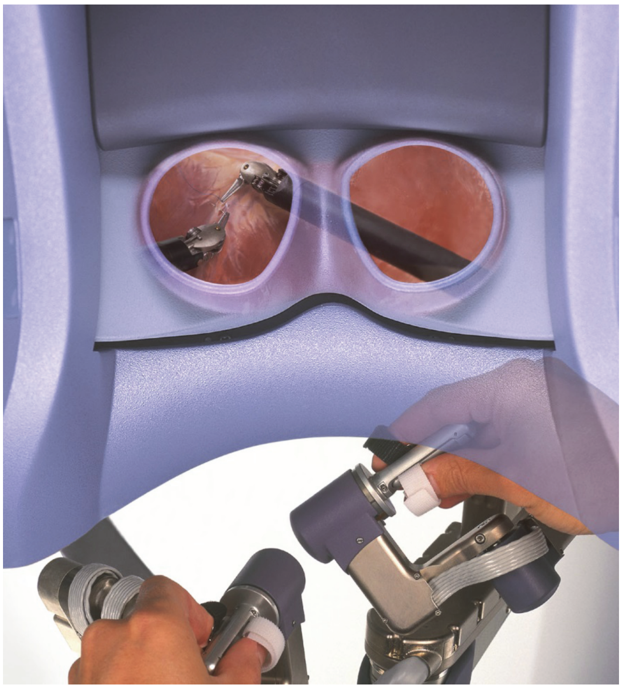

Figure 4. The surgeon console blends visualization and instrument control into an intuitive user interface (© 2017 Intuitive Surgical, Inc.).

The Encyclopedia of Medical Robotics Downloaded from www.worldscientific.com
by 5.61.122.207 on 03/25/22. Re-use and distribution is strictly not permitted, except for Open Access articles.

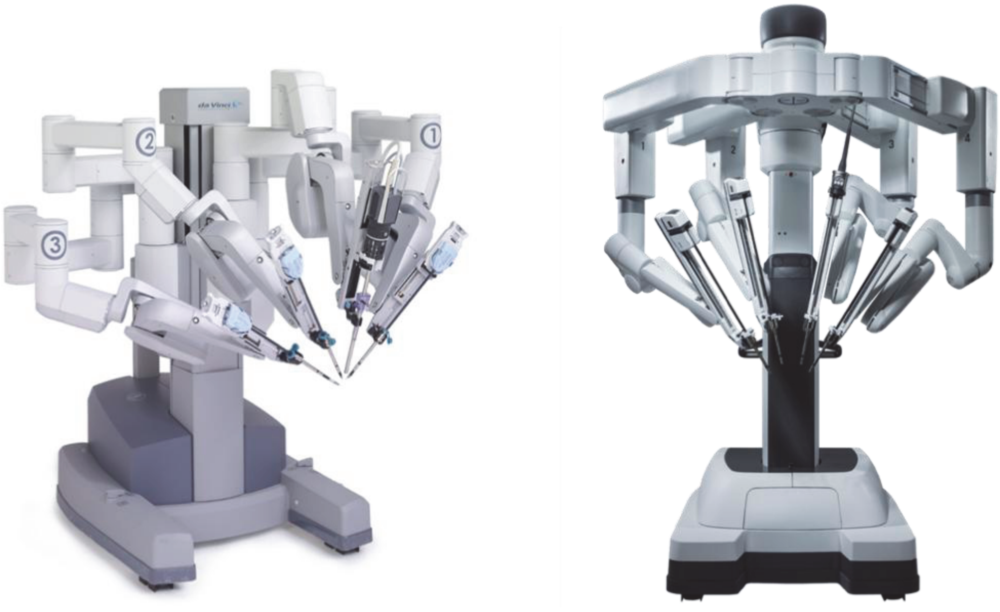

da Vinci Si Patient-side Cart *da Vinci* Xi Patient-side Cart

Figure 5. Comparison of the *da Vinci Si* and Xi setup structures (© 2017 Intuitive Surgical, Inc.).

areas of focus during the development of the *da Vinci* Xi® System were the roll-up, deployment and docking steps, with the intent to improve operative workflow, particularly for multi-quadrant procedures. This focus is evident in the redesigned kinematics of the setup structure for the *da Vinci* Xi System, as shown in Fig. 5.

The *da Vinci* Xi System uses a gantry system to position the instrument manipulators directly overhead the operating table. This gantry makes the position of the cart base largely independent from the orientation of the surgical workspace, thereby allowing the operating room staff more ease when positioning the base of the cart at the bedside, because they have fine control of the instrument cluster position and orientation overhead.

Contrast this with the *da Vinci Si*® patient-side cart, where the reachable workspace is highly dependent on the orientation of the cart. The team is required to anticipate a good location of the cart with respect to the patient, based on the requirements of the surgery, so as to avoid possible interruptions of the surgery for repositioning. This is just one example of a design solution that has been motivated by the need for ease of use and workflow efficiency.

In 2017, Intuitive Surgical released the *da Vinci* X™ Surgical System in order to provide a lower-cost solution to meet the needs of global customers who want a choice in price points, while offering access to some of the key innovations developed for the *da Vinci* Xi System. The *da Vinci* Xi System uses the same vision cart and surgeon consoles that are found on the *da Vinci* Xi System — which remains Intuitive Surgical's flagship platform — thus enabling customers the option of adding

The Encyclopedia of Medical Robotics Downloaded from www.worldscientific.com
by 5.61.122.207 on 03/25/22. Re-use and distribution is strictly not permitted, except for Open Access articles.

The Encyclopedia of Medical Robotics Downloaded from www.worldscientific.com
by 5.61.122.207 on 03/25/22. Re-use and distribution is strictly not permitted, except for Open Access articles.

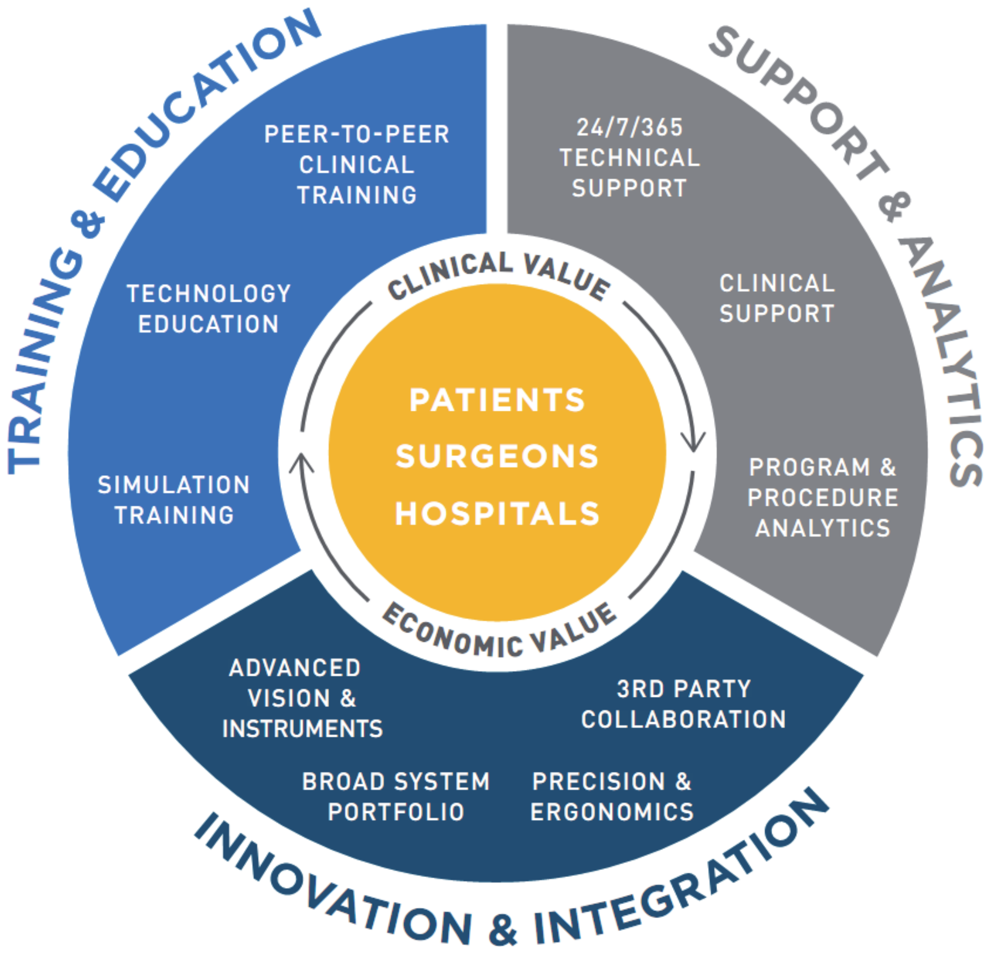

Figure 6. The Intuitive Surgical Ecosystem reflects all of the solutions, services and support offered to its customers. This begins with industry-leading surgical platforms, integrated tools and technologies, extends to comprehensive training and education, 24/7/365 support, and customized data and analytics.

advanced capabilities, and providing an upgrade pathway, should they choose to do so as their practice and needs grow. The System enables optimized, focused-quadrant surgery, such as for procedures like prostatectomy, partial nephrectomy, hernia repair, benign hysterectomy and sacrocolpopexy, among others. It features flexible port placement and state-of-the-art 3D digital optics, while incorporating many of the same advanced instruments and accessories as the *da Vinci* Xi System. The new system drives operational efficiencies by means of set-up technology that uses voice and laser guidance, drape design that simplifies surgery prep, and a lightweight, fully integrated endoscope.

While the mechatronic arms are the most visible part of the *da Vinci* System, building a robot-assisted surgery program requires an ecosystem of products and services. This ecosystem starts with a range of robotic systems that address different clinical needs and price points, as well as a family of dozens of different instruments and accessories. These include advanced instruments such as staplers and vessel sealers, as well as endoscopic stereo-imaging systems that include *Firefly*™ near-infrared imaging technology. The majority of *da Vinci* Systems are

The Encyclopedia of Medical Robotics Downloaded from www.worldscientific.com
by 5.61.122.207 on 03/25/22. Re-use and distribution is strictly not permitted, except for Open Access articles.

connected to a network infrastructure that allows Intuitive Surgical to perform predictive maintenance, minimize downtime, as well as to share analytic insights with customers. A global team of field service specialists provides rapid round-the-clock support for customer systems. Experienced surgeons teach over 40 different advanced courses to their peers in the use of *da Vinci* technology. More than 1,400 *da Vinci* Skills Simulators™ are in use, along with hundreds of real-time training consoles that support intraoperative, collaborative learning (dual console configuration) (Fig. 6). We discuss several of these aspects of the ecosystem in subsequent sections.

4. Visualization

The *da Vinci* Surgical System was among the first commercial products to use stereoscopic endoscopes to guide soft tissue surgery. Until recently, such endoscopes have captured and displayed white-light images that show only the visible surfaces of organs. This section introduces several advanced imaging technologies that can provide information that may not be directly visible in white-light images. For more detailed information, see Ref. [3].

4.1. *Fluorescence imaging*

Further innovation in visualization during robot-assisted surgical procedures is exemplified by near infrared fluorescence imaging. A fluorescent agent, a corresponding excitation light source, and a detector are required for fluorescent imaging as shown in Fig. 7. The surgical application of fluorescence imaging requires regulatory clearance for agents with demonstrated clinical utility and safety. The first

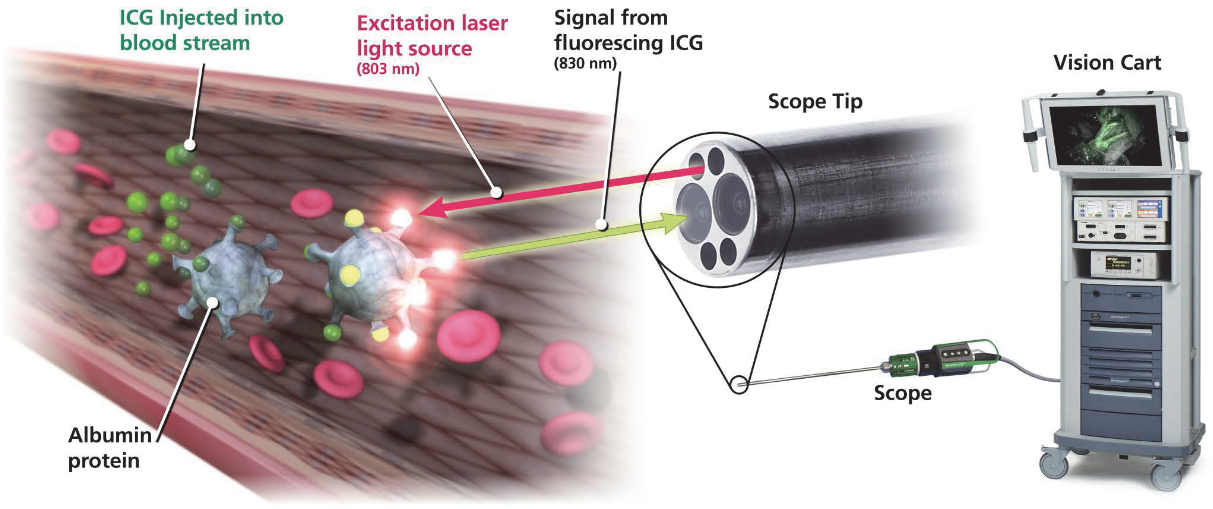

Figure 7. Overview of the *da Vinci* Firefly imaging feature. A laser light source is used to excite the fluorophores (at wavelengths around 800 nm) and the emitted light is captured by the image sensors on the endoscope (© 2017 Intuitive Surgical, Inc.).

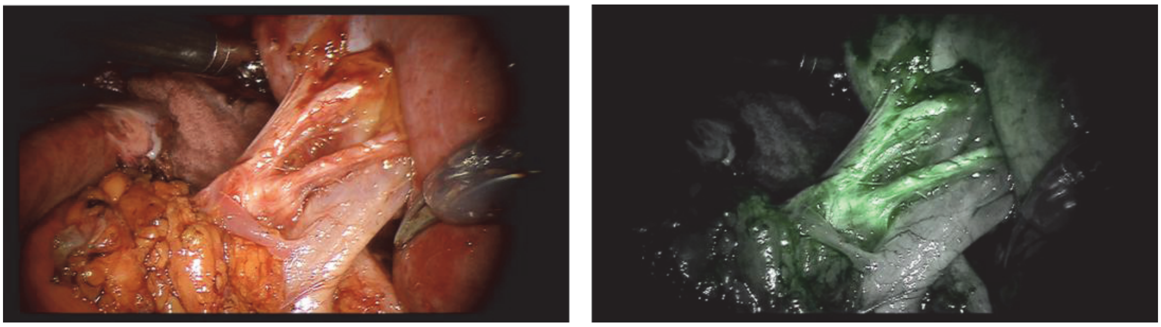

Figure 8. Vessel identification in *FireFly*® Fluorescence Imaging mode (view of renal hilum) during *da Vinci*® Partial Nephrectomy. A white light view is shown on the left and the FireFly view on the right (© 2017 Intuitive Surgical, Inc.).

agent to be widely used intraoperatively during robot-assisted surgical procedures is indocyanine green (ICG). Following injection into the bloodstream, ICG rapidly binds to plasma proteins in the blood. The near infrared signal that is detected by the imaging sensor in the endoscope is used to highlight the white light image with false color that provides the surgeon with an augmented view of the tissue, thereby giving the surgeon the ability to see vasculature and tissue perfusion.

The ICG is removed from the blood by the liver and is secreted into bile. This pathway for ICG excretion accounts for the rapid decrease in apparent brightness of ICG fluorescence in vasculature after administration in patients with healthy liver function. The ICG concentration in bile enables surgeons to image the bile duct structures, as illustrated in Fig. 8.[4] In general, the biliary structures become visible about 45 min following administration of ICG. The excitation and emission wavelengths of ICG are in the near infrared region of the light spectrum, and as adipose and fascia tissue are somewhat transparent at these wavelengths, it is possible to see the ICG fluorescence through a modest thickness of intervening tissue. Researchers continue to explore other applications of ICG and its application to conditions suited to robot-assisted surgery. There is ongoing research to explore the use of ICG to image lymphatic system drainage and to localize lymph nodes in order to reduce the invasiveness of node harvesting during cancer surgery. For a review of fluorescent imaging agents currently under development, see Ref. [5].

4.2. *Other optical imaging techniques*

While robot-assisted surgery has increased the proportion of surgical procedures performed minimally invasively, the opportunity to use optical signals to improve tumor identification and reduce adverse events can further benefit patients and the healthcare system. Many of these techniques can be combined with white light endoscopy. While some of these technologies are not necessarily specific to robot-assisted surgery, the inherent stability of robotic vision systems and the immersiveness of their user interfaces will enable easier integration. While still

The Encyclopedia of Medical Robotics Downloaded from www.worldscientific.com
by 5.61.122.207 on 03/25/22. Re-use and distribution is strictly not permitted, except for Open Access articles.

limited in early stages, it is likely that these multi-spectral vision-based techniques will eventually provide surgeons with the ability to more clearly discern tissue structures and pathologies, thus enabling improved patient care.

Multi-spectral imaging can be used to acquire and analyze data across the electromagnetic spectrum. One example of this — Narrow Band Imaging (NBI) — uses selective bands of red, blue and green illumination to better visualize topical tissue features. As an inherently surface-based technology, NBI shows promise to help evaluate suspicious superficial lesions in the head and neck, as well as bladder cancers.[6]

Many spectral-based technologies cannot easily be applied to current robot-assisted robotic surgical procedures due to their limited field of view. Robotics can make the acquisition of image mosaics easier, as acquisition speed and instrument travel over tissue can be controlled. Image stitching (mosaicking) has been applied to Mauna Kea's Cellvizio® microendoscope, which traditionally offers a field of view in the hundreds of microns range (Mauna Kea Technologies, Paris, France). Similar field-of-view challenges exist for optical coherence tomography techniques (OCT), such that a mosaicking approach may also be helpful for visualization of this imaging modality during surgery.

4.3. *Tomographic imaging*

Tomography refers to imaging cross-sections of an object using penetrating waves such as X-rays (e.g., computed tomography, CT), gamma-rays (e.g., single photon emission computed tomography, SPECT), radio-frequency waves (e.g., magnetic resonance imaging, MRI), mechanical waves (e.g., ultrasound), etc. Tomographic imaging provides sectional images, which can provide information beyond the surface of tissue, as opposed to reflective images captured by endoscopes or bare eye vision.

Robot-assisted surgery for soft tissue has mostly focused on reflective imaging that allows the user to see only the visible surface of organs. As described above, fluorescence imaging has been used to provide subsurface information, for example in colorectal surgery.[7] Although tomographic imaging is not currently an integral part of soft tissue robot-assisted surgical systems, it is often used to visualize deep tissue structures such as solid tumors, vasculature, etc., prior to surgery.

As suggested in the Refs. [8] and [9], use of tomographic images during robot-assisted surgery may result in increased accuracy and speed, fewer complications by avoiding critical structures, and reduced chance of cancer recurrence by more accurate tumor resection and better lymphadenectomy under image guidance.[8,9,b]

[b]This study was performed to validate the feasibility and role of image-guided robotic surgery using pre-operative computed tomography (CT) images for the treatment of gastric cancer.

The Encyclopedia of Medical Robotics Downloaded from www.worldscientific.com
by 5.61.122.207 on 03/25/22. Re-use and distribution is strictly not permitted, except for Open Access articles.

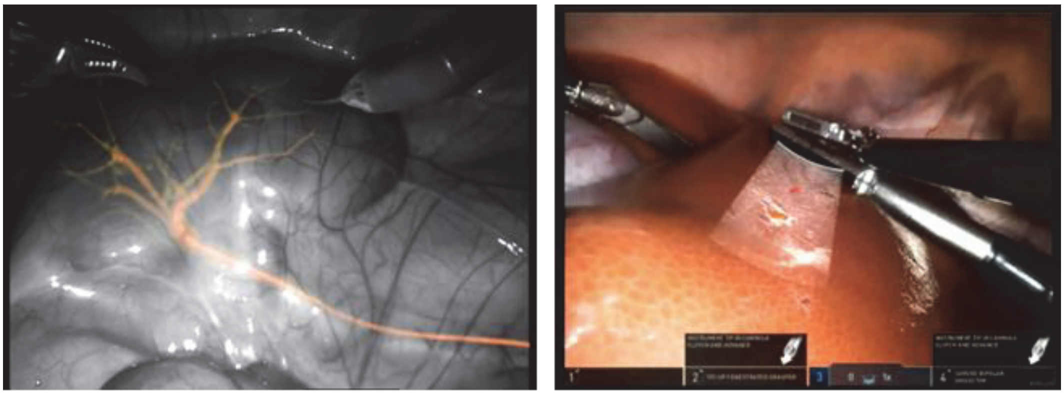

(a) (b)

Figure 9. (a) overlay of renal arteries from a rendered CT angiogram over grayscale endoscopic images and (b) overlay of ultrasound images on endoscopic images (© 2017 Intuitive Surgical, Inc.).

Tomographic images are often acquired pre-operatively and need to be aligned (registered) to intraoperative patient coordinates — this is particularly challenging within soft tissue structures, due to complex tissue deformations.[8]

Intraoperative imaging modalities such as ultrasound are used to acquire live images during surgery. The *da Vinci* System supports feeds from auxiliary video streams into the surgical display (TilePro), thus allowing third-party image and video sources to be viewed adjacent to live endoscopic video. Although the TilePro feature can be used to display tomographic images intraoperatively, the lack of automated alignment between endoscopic view and tomographic view makes it counter-intuitive to use tomographic images to guide the surgical procedure. Numerous research efforts have attempted to solve the hand–eye coordination problem in order to make more effective use of tomographic imaging modalities with the *da Vinci* System.[10] Figure 9 shows examples of augmented reality images that combine endoscopic and tomographic images.

5. Tissue Interaction

A family of instruments has been developed for the *da Vinci* System in order to facilitate tissue manipulation in various types of surgical procedures. Many of these instruments have an articulated wrist mechanism to allow for dexterous and intuitive tissue interaction, following the surgeon's wrist articulation while controlling motion from the master interfaces of the surgical console. *EndoWrist*® is the trade name for these articulated instruments, which include various types of scissors, forceps, needle drivers, retractors, monopolar and bipolar energy instruments, stabilizers, staplers, and vessel sealers. In this section, we review three of the most advanced instruments: stapler, vessel sealer, and Harmonic Ace.

The Encyclopedia of Medical Robotics Downloaded from www.worldscientific.com
by 5.61.122.207 on 03/25/22. Re-use and distribution is strictly not permitted, except for Open Access articles.

5.1. *Stapler*

The *EndoWrist* Stapler is an articulated surgical device intended to be used with the *da Vinci Si* and Xi Systems for resection, transection, or creation of anastomosis in general, thoracic, gynecologic, and urologic surgery. It allows surgeons to control the positioning and firing of a reload (staple cartridge) that places multiple staggered rows of staples and transects the tissue with a knife blade. The result is a clean division of tissue with no bleeding. The stapler instrument can be reloaded multiple times with stapler reloads — these are disposable and contain both staples and a knife blade.

The Stapler instrument — as shown in Fig. 10 — has two opposing jaws and six DoFs: roll, pitch, yaw, grip, "clamp," and "fire". Roll, pitch, yaw, and grip are used to position the upper and lower jaws of the instrument relative to the target tissue and are controlled in the same manner as other *EndoWrist* instruments, via master manipulators on the surgeon console. The term "clamp" is the same motion as grip, but uses a different mechanism to provide significantly higher grip force. The term "fire" describes the combined action of implantation of staples and transection (translating blade) of the target tissue. Both functions are activated and controlled by the foot pedals at the surgeon console. The lower jaw of the instrument houses the staple reload, the upper jaw contains features which "form" the staples such that they remain implanted in the tissue.

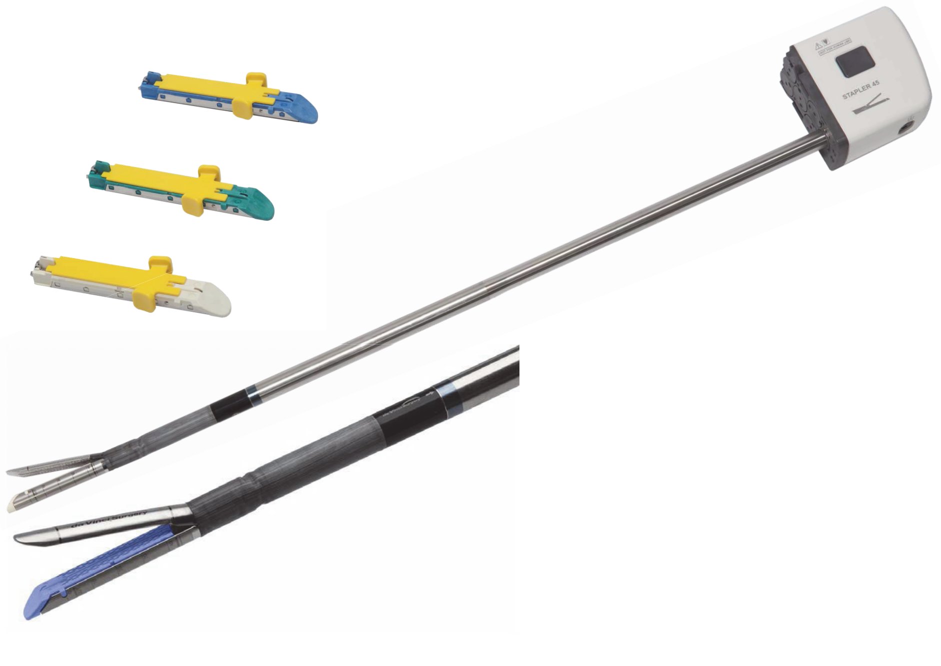

Figure 10. The *da Vinci* Xi EndoWrist Stapler 45 with three different stapler reloads shown on top left (from top to bottom: blue, green, and white); illustration of the instrument shown in the middle, and the distal part of a loaded stapler at the bottom (© 2017 Intuitive Surgical, Inc.).

The Encyclopedia of Medical Robotics Downloaded from www.worldscientific.com
by 5.61.122.207 on 03/25/22. Re-use and distribution is strictly not permitted, except for Open Access articles.

The Encyclopedia of Medical Robotics Downloaded from www.worldscientific.com
by 5.61.122.207 on 03/25/22. Re-use and distribution is strictly not permitted, except for Open Access articles.

5.2. *Vessel sealer*

The *da Vinci EndoWrist One* Vessel Sealer — shown in Fig. 11 — is a disposable advanced bipolar cautery instrument that seals and cuts vessels up to 7 mm in diameter. By applying precise pressure and controlled energy delivery, soft tissue proteins denature within the range of 60−90°C, hence the inside wall of the vessel is melted (or fused) together. The energy delivery is controlled based on tissue impedance measurement during sealing, so as to maintain temperature within a range that results in sealing rather than charring or burning. Once sealed, the vessel can be transected by firing a mechanical knife that moves along the length of the instrument jaws, in a slot through the center of the electrodes.

5.3. *Harmonic ace*

High-power (10–1000 W/cm^2), low-frequency (20–100 kHz) ultrasonic vibration has a wide range of applications in medical procedures, such as dentistry, phacoemulsification (cataract surgery), maxillofacial surgery, neurosurgery, tissue dissection and tumor fragmentation, as well as vascular plaque ablation. Intuitive Surgical has collaborated with Ethicon Endo-Surgery (Somerville, NJ, USA) to offer

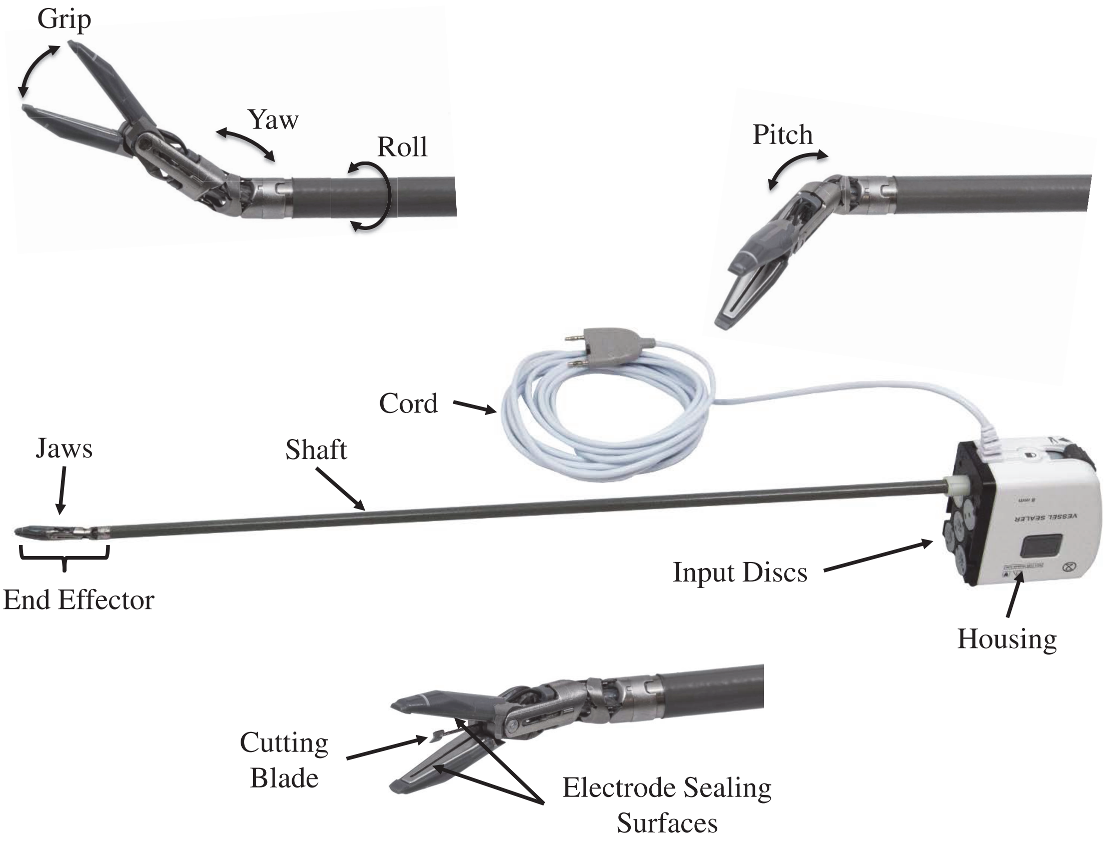

Figure 11. *da Vinci* Vessel Sealer instrument (© 2017 Intuitive Surgical, Inc.).

a robot-assisted ultrasonic scalpel. The resulting instrument, the Harmonic Ace, is a disposable instrument that may be used for

- dissection, cutting, grasping and manipulating tissue,
- otomy creation (cutting into an organ using tip of the blade),
- back-cutting of soft tissue (using the outer edge of the blade to cut soft tissue, rather than cutting between the jaws).

The Encyclopedia of Medical Robotics Downloaded from www.worldscientific.com
by 5.61.122.207 on 03/25/22. Re-use and distribution is strictly not permitted, except for Open Access articles.

5.4. *Integrated table motion*

The integrated table motion feature of the *da Vinci* Xi System (Fig. 12) allows the system to communicate with TRUMPF Medical's advanced operating table — the TruSystem® 7000dV (TRUMPF Medezin Systeme, Saalfeld, Germany). With this feature, surgical teams can reposition the operating table during a procedure, while the robotic arms remain docked and while the surgeon maintains control of instruments, based on computer-controlled, coordinated motion between the *da Vinci* System and table. The table and the surgical system are synchronized, so that the surgical system adjusts the gantry and instrument arms to maintain the pose of instruments and endoscope relative to the patient's anatomy while the table moves (Fig. 13). This feature can be used to

- optimally position the table so that gravity exposes anatomy during multi-quadrant procedures;
- maximize reach and access to target anatomy, thus enabling surgeons to interact with tissue at an ideal working angle; and
- reposition the table during the procedure to enhance the anesthesiologist's care of the patient.

Several key functions make this feature safe and effective:

- **Port Following** — The arms and gantry seamlessly follow the ports to leverage the *da Vinci* Xi System's full range of motion.
- **Center of Motion** — Combines table Trendelenburg and Slide to move the patient around a virtual pivot point; maximizes the range of motion.
- **Remote Center Monitoring** — Redundant software checks ensure cannulas and arms move in unison for added safety.
- **Intuitive Instrument Control** — Surgeon maintains control from the surgeon console while the table is moving.
- **Table Tracking** — Actively rotates the *da Vinci* Xi camera and instruments to maintain a consistent orientation to anatomy during table movement.

The Encyclopedia of Medical Robotics Downloaded from www.worldscientific.com
by 5.61.122.207 on 03/25/22. Re-use and distribution is strictly not permitted, except for Open Access articles.

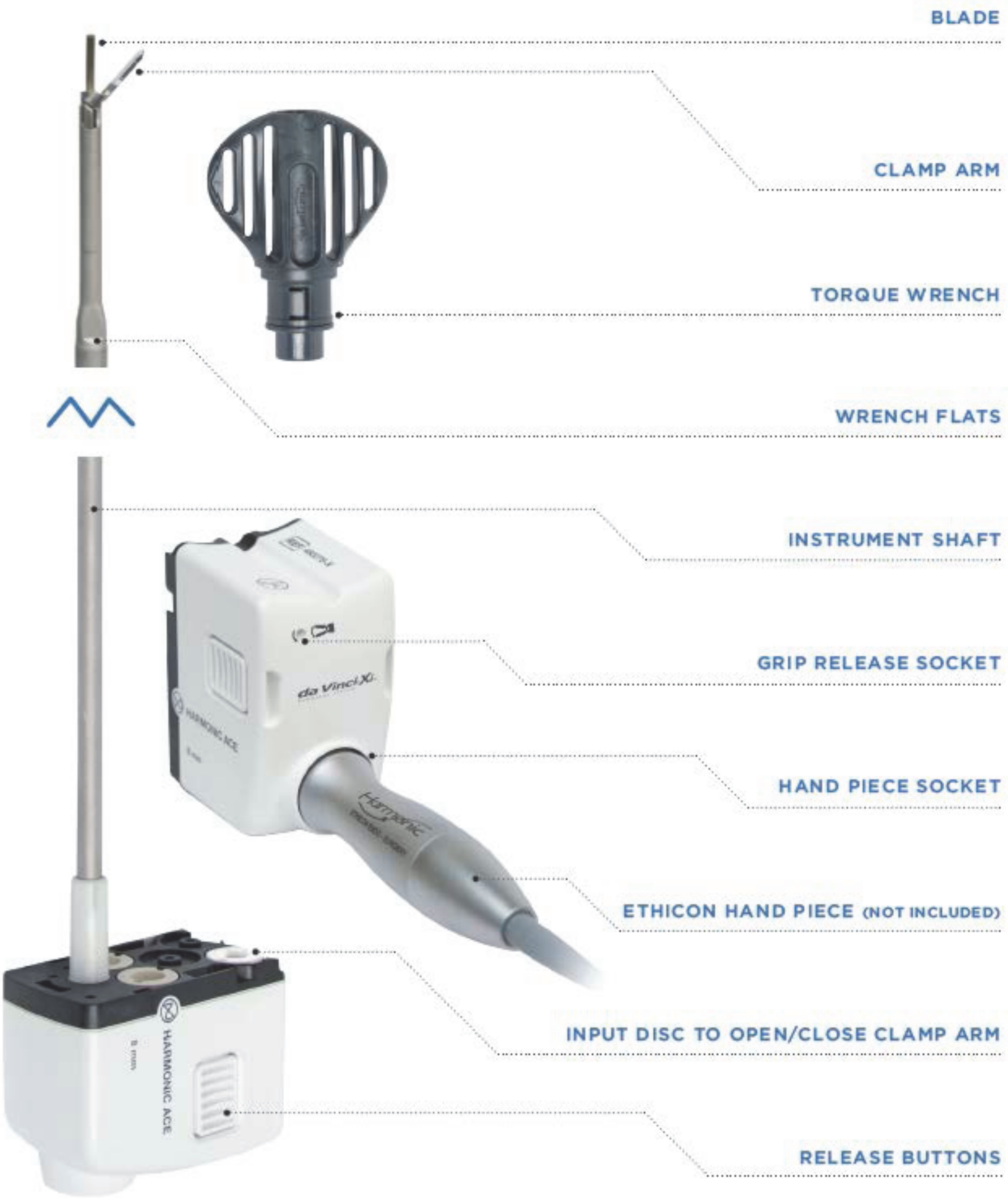

Figure 12. Harmonic Ace instrument for *da Vinci* Xi. A reusable Ethicon Endo-Surgery handpiece must be assembled to the disposable Harmonic Ace instrument. Handpiece itself is then electrically powered by an Ethicon generator (© 2017 Intuitive Surgical, Inc.).

Prior to the introduction of this feature, any intraoperative changes in table position or orientation required a pause in the surgical procedure in order to undock the robotic arms, reposition the bed, and then redock and resume. Therefore, the integrated table motion feature improves efficiency in the operating room, while offering an additional way of managing surgical access, exposure, and reach — essentially by employing gravity as a fourth "invisible instrument."

The Encyclopedia of Medical Robotics Downloaded from www.worldscientific.com
by 5.61.122.207 on 03/25/22. Re-use and distribution is strictly not permitted, except for Open Access articles.

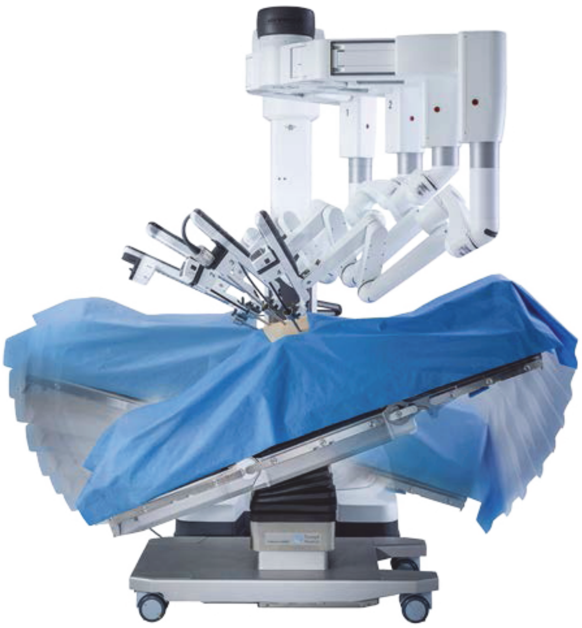

Figure 13. Integrated operating table motion with the *da Vinci* Xi Surgical System (© 2017 Intuitive Surgical, Inc.).

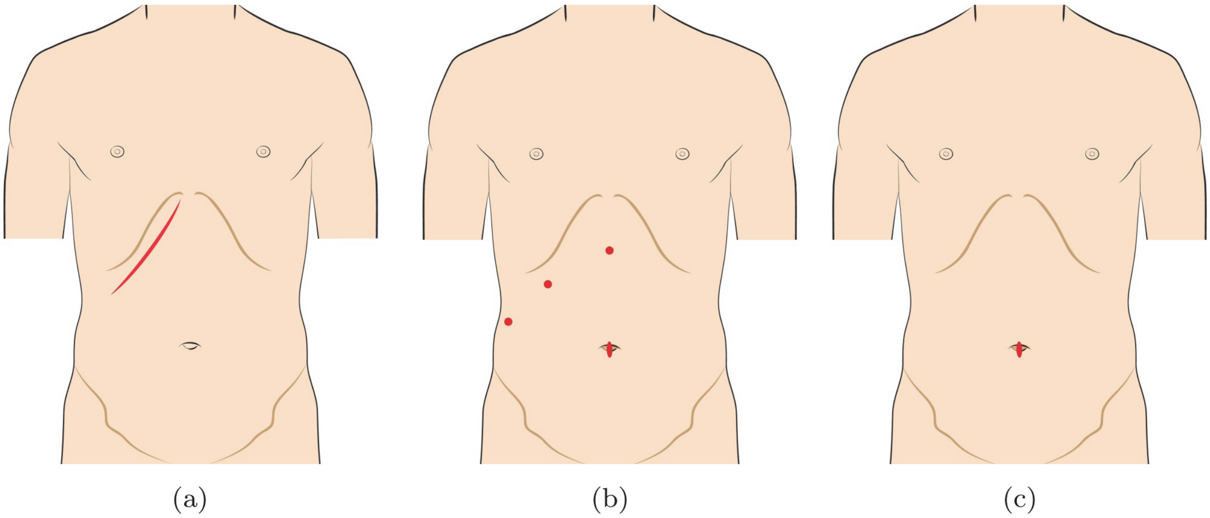

Figure 14. (a) Open surgery with 5–8 inches of incision, (b) multi-port laparoscopic surgery with 4 small incisions, and (c) single-site or single-port surgery with only one incision.

6. Surgical Access

The *da Vinci* System provides multiple ports of surgical access through small incisions in the patient's body. This may reduce the morbidity of the procedure, when compared to open surgery where large incisions are required to provide access.[11] Invasiveness may be further reduced by using only one incision or by accessing the body via natural orifices. As an example, Fig. 14 shows incisions required for open, multi-port and single-port laparoscopic cholecystectomy (gallbladder removal) procedures.

The Encyclopedia of Medical Robotics Downloaded from www.worldscientific.com
by 5.61.122.207 on 03/25/22. Re-use and distribution is strictly not permitted, except for Open Access articles.

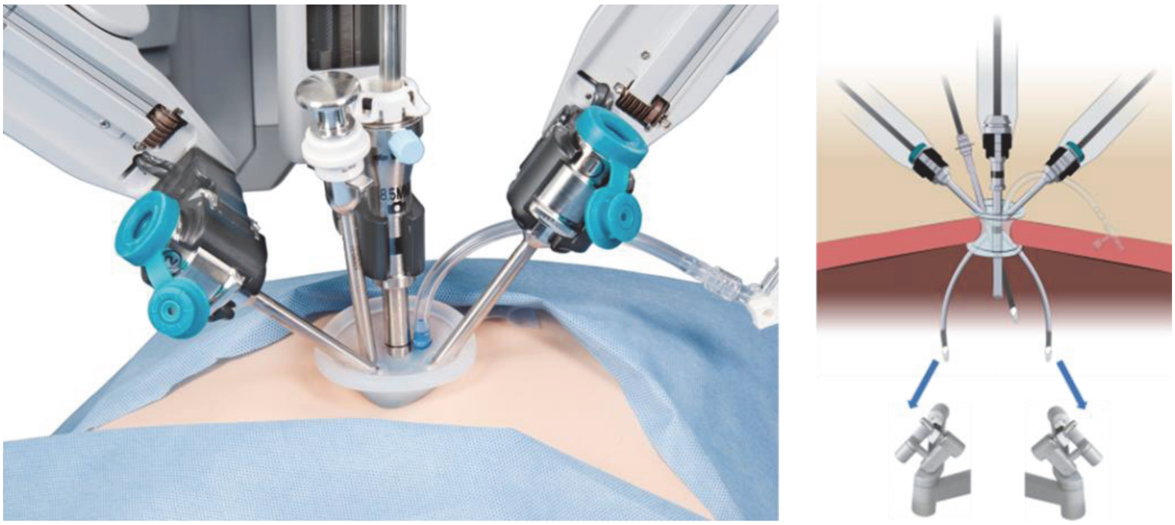

(a) (b)

Figure 15. *da Vinci Si* Single-Site System: (a) Patient-side cart setup with three arms and curved cannulas used with flexible instruments and (b) illustration of master manipulator associations with Single-Site instruments (© 2017 Intuitive Surgical, Inc.).

6.1. *da Vinci Single-Site technology*[c]

The *da Vinci* Single-Site technology enables surgeons to perform single incision surgery with the *da Vinci* platform (Si, Xi, and X). Currently, *da Vinci* Single-Site is used for cholecystectomy, hysterectomy and other types of procedures in abdominal and pelvic areas. Five millimeter semi-rigid instruments are flexible enough to curve through the cannula (as shown in Fig. 15), but still rigid enough to manipulate tissue and to provide retraction.

6.2. *da Vinci SP® System*[d]

The *da Vinci* SP single-port system — under development and not yet commercially available (as of June 2017) — introduces articulated instruments and an articulated camera through a single incision. This design allows all instruments to enter a single cannula along parallel axes, as opposed to single-site technology on the multi-port systems. The snake-like articulated instruments and camera can be manipulated independently to provide surgical access after entering the body. This will make *da Vinci* SP System suitable for procedures that require natural orifice access, such as trans-oral, trans-rectal and trans-vaginal procedures.

---

[c] As of June 2017, the *da Vinci* Single-Site technology may not be available in all markets for all type of procedures pending individual country approvals (e.g., Product is not 510(k) Cleared for abdominal procedures).
[d] In the U.S. (as of June 2017), the *da Vinci* SP System is not 510(k) Cleared.

7. Technology Training

Each successive generation of the *da Vinci* System offers the surgical team improved ease of use in addition to improved features. Although training requirements vary from institution to institution, and different trainings are offered by various sources, training and continuing education for the surgeon and surgical staff remain crucial for successful use of the system. The nature of the *da Vinci* System presents both challenges and unique opportunities for training clinicians.

Perhaps, the greatest challenge to training new *da Vinci* users is access to the system. In hospitals where the *da Vinci* System is highly utilized, the system has little idle time to use for personnel training. Even if the system is not being used, the operating room that houses it may be in use for other procedures, rendering the *da Vinci* System unavailable. The result is that training opportunities are often limited to evenings and weekends. Availability of *da Vinci* instruments and accessories can also pose a challenge. Finally, coordinating the availability of the operating room, system, and accessories with the schedules of busy surgeons and surgical staff means that training time is often extremely limited and therefore must be used thoughtfully and efficiently (Fig. 16). The computer-assisted nature of the *da Vinci* System creates unique opportunities to deliver enhanced training experiences to surgeons and staff. The central computing system mediates all of the user commands and system outputs. An obvious application of this information stream.[12] is to measure and evaluate how the user is operating the system[13–15] such approaches could eventually be used for intraoperative feedback to the users during surgical procedures as well as during training sessions. Another application of the information stream is to replace the endoscopic video feed with a virtual reality feed to the surgeon console; indeed, the dynamics and controls of the entire patient side cart can be computationally

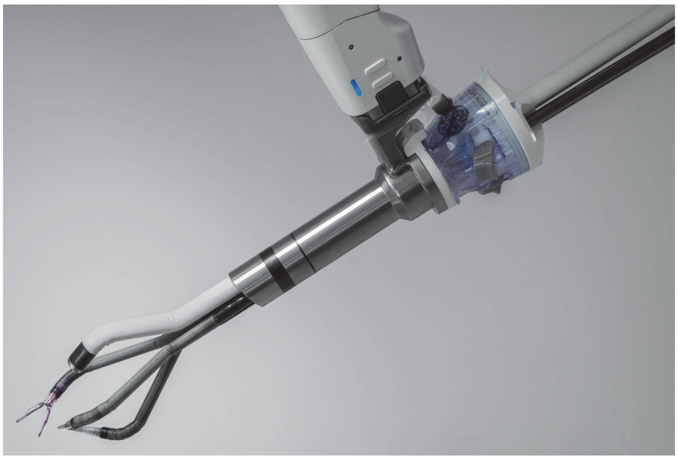

Figure 16. The *da Vinci* SP System, single-port cannula, articulated instruments and camera shown (© 2017 Intuitive Surgical, Inc.).

The Encyclopedia of Medical Robotics Downloaded from www.worldscientific.com
by 5.61.122.207 on 03/25/22. Re-use and distribution is strictly not permitted, except for Open Access articles.

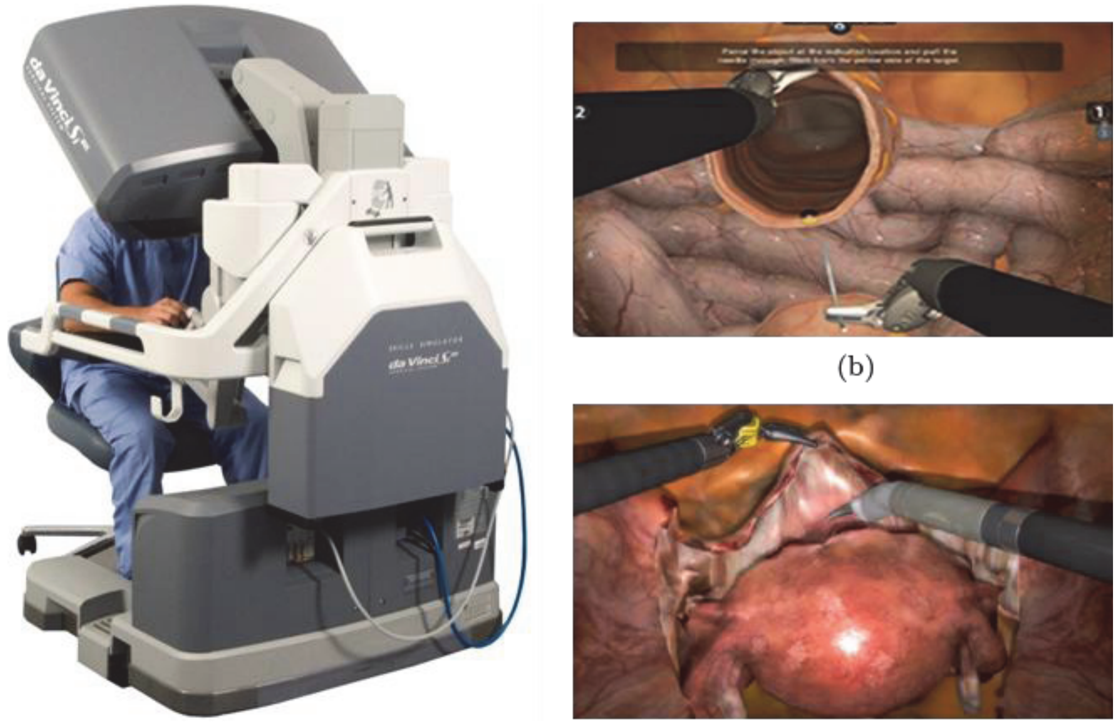

(a) (c)

Figure 17. The *da Vinci* Skills Simulator, shown mounted on the surgeon console: (a) Examples of digital training content available on the Skills Simulator. (b) Image demonstrates a needle targeting exercise (courtesy of Mimic Technologies, Inc., Seattle, WA). (c) Image demonstrates a simulated hysterectomy procedure (courtesy of 3D Systems, Inc., Rock Hill, SC) (© 2017 Intuitive Surgical, Inc.).

simulated and fed back to the surgeon console. Intuitive Surgical's *da Vinci* Skills Simulator (Fig. 17) does just that, and it enables the surgeon to practice using the console without the accessories and support staff. The opportunities for virtual reality simulation in minimally invasive surgical training in general, and *da Vinci* training in particular,[16–19] have been extensively documented, and include quantitative metrics, unlimited diversity of training and surgical scenarios, and the future potential for patient-specific procedural rehearsal.[20–22]

da Vinci Systems can be accessed by Intuitive Surgical via a secure network connection to facilitate preventative maintenance and customer service technical support. Network connectivity can also be used to support telementoring, for surgeons who may be either mentors or students of the surgeon operating at the console. The *da Vinci Connect* technology supports two-way audio communication and a 2D endoscopic view for the remote surgeon.[23,24] The platform also allows the remote surgeon to "draw" on the surgeon's console screen. Building on this technology, a new telepresence platform[25] will enable greater interactivity by providing the remote surgeon with a 3D endoscopic view and the ability to manipulate virtual instruments that appear in the console surgeon's field of view. While the actual instruments always remain in control of the surgeon sitting at

The Encyclopedia of Medical Robotics Downloaded from www.worldscientific.com
by 5.61.122.207 on 03/25/22. Re-use and distribution is strictly not permitted, except for Open Access articles.

the console, this allows the remote surgeon to demonstrate optimal instrument use through the visual overlay.

The benefits of computer-assisted simulation could be extended to training the surgical staff as well. Efficiently setting up the system prior to a procedure is important for keeping the operating room running on schedule, and requires knowledge of how to perform individual tasks as well as how to coordinate all of the tasks as a team.

It is important that the development of training tools be driven by learner needs, rather than technological novelty. As robotic-assisted surgery gains broader adoption, the types of learners and their needs grow as well. Thoughtful attention to different learners' needs when developing training content and training technologies will ensure that impactful and efficient learning is available to all *da Vinci* users.

8. Clinical Adoption

Intuitive Surgical's current product lines focus on five surgical specialties: gynecologic surgery, urologic surgery, general surgery, cardiothoracic surgery, and head and neck surgery. Specific clearances vary across regulatory bodies worldwide and across the various models of *da Vinci* Systems. In the United States, the *da Vinci* System is classified by the FDA as a Class II device, and as such clearances are made for a specific set of indications. At the time of writing, these clearances include indications for urologic surgical procedures, general laparoscopic surgical procedures, gynecologic laparoscopic surgical procedures, (specifically for the *da Vinci Si* System only: trans-oral otolaryngology surgical procedures restricted to benign and malignant tumors classified as T1 and T2 and for benign base of tongue resection procedures), general thoracoscopic surgical procedures, and thoracoscopically assisted cardiotomy procedures. The system can be employed with adjunctive mediastinotomy to perform coronary anastomosis during cardiac revascularization. The system is indicated for adult and pediatric use except for trans-oral otolaryngology surgical procedures.

A goal within some of these procedures and specialties is to drive the conversion of open surgery to MIS and to facilitate difficult minimally-invasive operations in order to approach the surgical ideals described in Section 1.1.

8.1. *Procedure trends*

In 2015, total U.S. procedure volume was approximately 499,000, of which 20% was in urology, 48% was in gynecology, and 28% was in general surgery. Procedure volume from markets outside of the U.S. was approximately 153,000 during that same year, of which most procedures were in urology. Procedure trends between 2011 and 2016 are illustrated in Fig. 18.

The Encyclopedia of Medical Robotics Downloaded from www.worldscientific.com
by 5.61.122.207 on 03/25/22. Re-use and distribution is strictly not permitted, except for Open Access articles.

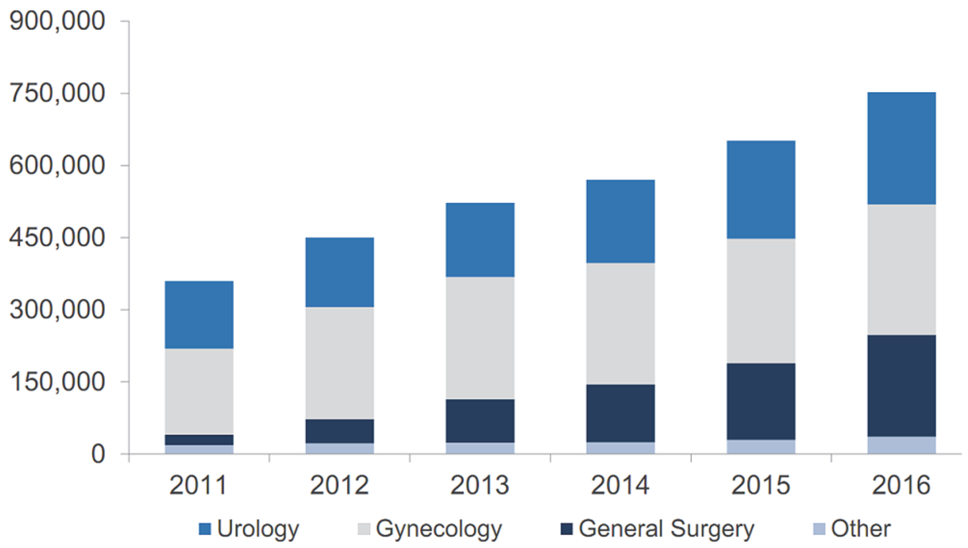

Figure 18. Worldwide *da Vinci* procedure growth from 2011 to 2016.

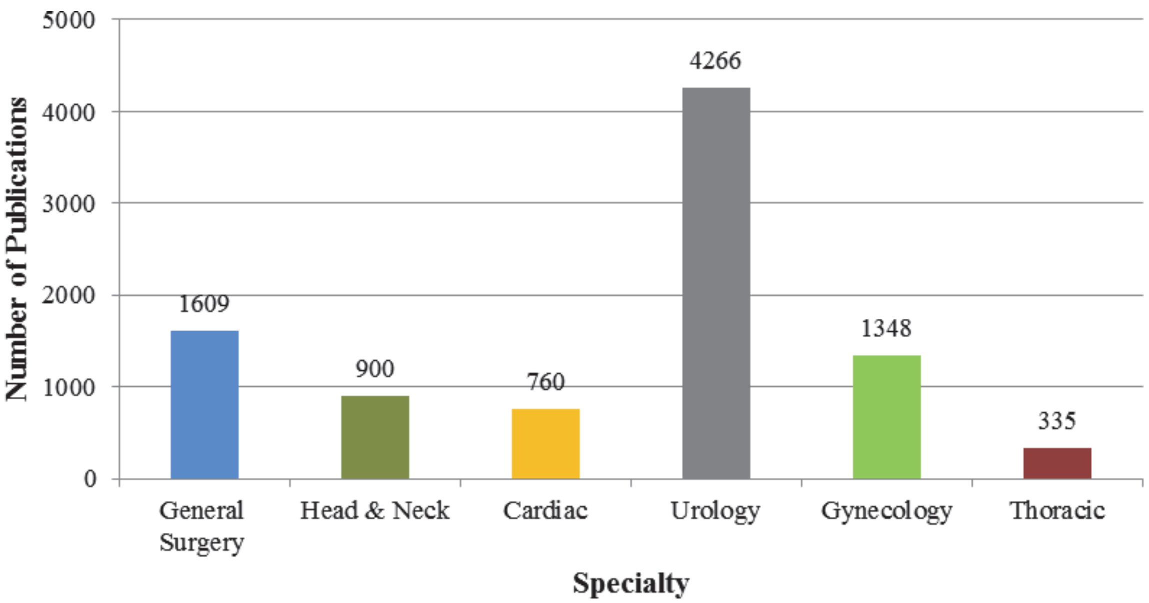

Figure 19. Publications on *da Vinci* technology from 1998 to 2015 by surgical specialty.

8.2. *Publications*

As public agencies seek to understand the impact of new technologies on health-care outcomes and costs, peer-reviewed clinical publications and evidence-based medicine have become increasingly important. There are currently over 11,000 PubMed-indexed publications across multiple surgical specialties related to the clinical uses of the *da Vinci* System, the vast majority of which were researched and written independent of Intuitive Surgical. Figure 19 shows publications by surgical specialty from 1998 to 2015. While some critics cite the lack of clinical evidence for the efficacy of robotic surgery, the peer-reviewed literature is both deep and compelling across many clinical applications of robotics.

The Encyclopedia of Medical Robotics Downloaded from www.worldscientific.com
by 5.61.122.207 on 03/25/22. Re-use and distribution is strictly not permitted, except for Open Access articles.

9. Conclusions and Future Opportunities

At Intuitive Surgical, we are keenly focused on continuing to enhance the value of our product and service ecosystem for our customers by striving to make surgery more effective, less invasive and easier on surgeons, patients and their families. This chapter has described the *da Vinci* System as a platform technology that we can leverage to further enhance surgeon perception, tissue manipulation, and minimally invasive access to diseased tissue.

In the area of visualization, two major trends of research and development may shape the future of image guidance in robot-assisted surgery: (a) improvements in visualization techniques mainly driven by the gaming industry. In a recent survey of robotic urology surgeons, 87% felt that there is a role for augmented reality as a navigational tool in robot-assisted surgery[26] and (b) advances in molecular imaging are likely to find more use in robot-assisted surgery with the advent of molecular markers specific to various tissue types and pathologies.[27]

In the area of tissue interaction, mechanical manipulation of soft tissue using instruments such as scissors, forceps and scalpels has been practiced for centuries and is still in use in operating rooms. The first use of electrosurgery at the Brigham and Women's Hospital (Boston, Massachusetts) dates back to 1926. Development of advanced energy instruments that use electric currents, ultrasonic vibrations, lasers for cutting, tissue fusion and welding, etc. has been a major trend in the past few decades and we anticipate further advancements in energy instruments that are more efficient and precise. Robotic platforms will enable greater dexterity and control of these instruments.

In terms of minimally invasive access to tissues, five major areas of future development in surgical access can be identified: (a) further miniaturization of surgical instruments, (b) increased use of endoluminal or percutaneous access driven by advances in snake robot-assisted technologies, (c) swallowable robots,[28] (d) targeted therapy with magnetic guidance,[29] and (e) non-invasive access via focal therapy.[30,e]

Autonomous surgery is currently the subject of scientific research and is likely to be a topic of debate as regulatory challenges are realized. In soft tissue surgeries, the anatomy can move in complex ways, for example, due to instrument interactions, respiration, and insufflation. Current systems rely on the surgeon's ability to recognize and track tissue motions and changes during surgery and to command instrument motion. As technologies advance, humans will likely remain in the loop for the foreseeable future; however, supervised automation of certain surgical tasks or subtasks is an active area of research, with potential for clinical

[e]A prototype magnetic resonance image (MRI)-conditional robot was developed for navigating a high-intensity focused ultrasound (HIFU) system in order to treat prostate cancer transrectally.

The Encyclopedia of Medical Robotics Downloaded from www.worldscientific.com
by 5.61.122.207 on 03/25/22. Re-use and distribution is strictly not permitted, except for Open Access articles.

The Encyclopedia of Medical Robotics Downloaded from www.worldscientific.com
by 5.61.122.207 on 03/25/22. Re-use and distribution is strictly not permitted, except for Open Access articles.

adoption in future.[31] This may include gradual introduction of guidance and warning features that will require the system to have some knowledge of the surgical task, similar to early aspects of autonomy in automobiles, where the first steps were recognition of road markings, obstacles, cars and pedestrians. Machine learning and advanced data analytics are likely to introduce new ways to support the surgical team by augmenting human capabilities with emerging information processing and computational capabilities. This will no doubt continue to extend the partnership between humans and machines in order to improve clinical outcomes.

We look forward to the innovations that are yet to be imagined and built, in order to power the next revolution in surgery. Multi-disciplinary collaboration has clearly been a key component of the development of our field to date and will become increasingly important as new capabilities are realized and new applications are explored. This teamwork between clinical scientists, surgeons, academic researchers, industry engineers, regulatory groups, and many others will help to transition novel ideas into technologies that will ultimately benefit patients and their families in remarkable new ways.

References

1. J. Rosen, B. Hannaford *et al.*, *Surgical Robotics: Systems Applications and Visions* (Springer Science and Business Media, 2011).
2. R. A. Heinlein, (1969). *Waldo, and Magic, Inc.* (Alan G. Gates).
3. M. Azizian, I. McDowall *et al.*, Visualization in Robotic Surgery, *The SAGES Atlas in Robotic Surgery* (2017) in press.
4. G. Spinoglio, F. Priora *et al.*, Real-time near-infrared (NIR) fluorescent cholangiography in single-site robotic cholecystectomy (SSRC): A single-institutional prospective study, *Surg. Endosc.* **27**(6), 2156–2162 (2013).
5. J. Sorger, ed. *Clinical Milestones in Optical Imaging*. Imaging and Visualization in the Modern Operating Room: A Comprehensive Guide for Physicians, Springer (2015).
6. C. Zheng, Y. Lv *et al.*, Narrow band imaging diagnosis of bladder cancer: Systematic review and meta-analysis, *BJU Int.* **110**(11b), E680–E687 (2012).
7. M. Hellan, G. Spinoglio *et al.*, The influence of fluorescence imaging on the location of bowel transection during robotic left-sided colorectal surgery, *Surg. Endosc.* **28**(5), 1695–1702 (2014).
8. S. D. Herrell, D. M. Kwartowitz *et al.*, Toward image guided robotic surgery: System validation, *J. Urol.* **181**(2), 783–790 (2009).
9. Y. M. Kim, S.-E. Baek *et al.*, Clinical application of image-enhanced minimally invasive robotic surgery for gastric cancer: A prospective observational study, *J. Gastrointest. Surg.* **17**(2), 304–312 (2012).
10. J. Leven, D. Burschka *et al.*, DaVinci canvas: A telerobotic surgical system with integrated, robot-assisted, laparoscopic ultrasound capability, *Medi. Image Computing and Computer-Assisted Intervention — MICCAI 2005* (Springer, 2005), pp. 811–818.
11. J. E. Anderson, D. C. Chang *et al.*, The first national examination of outcomes and trends in robotic surgery in the United States, *J. Am. College Surg.* **215**(1), 107–114 (2012).
12. S. DiMaio and C. Hasser, The da Vinci research interface, miccai workshop on systems and architectures for computer assisted interventions, *MIDAS Journal* (2008). http://hdl.handle.net/10380/1464.

13. H. C. Lin, I. Shafran *et al.*, Automatic detection and segmentation of robot-assisted surgical motions, *Med. Image Comput. Assist. Interv.* **8**(Pt 1), 802–810 (2005).
14. H. C. Lin, I. Shafran *et al.*, Towards automatic skill evaluation: Detection and segmentation of robot-assisted surgical motions, *Comput. Aided. Surg.* **11**(5), 220–230 (2006).
15. R. Kumar, A. Jog *et al.*, Objective measures for longitudinal assessment of robotic surgery training, *J. Thorac. Cardiovasc. Surg.* **143**(3), 528–534 (2012).
16. H. Abboudi, M. S. Khan *et al.*, Current status of validation for robotic surgery simulators — A systematic review, *BJU Int.* **111**(2), 194–205 (2013).
17. J. D. Bric, D. C. Lumbard *et al.*, Current state of virtual reality simulation in robotic surgery training: A review. *Surg. Endosc.* **30**(6), 2169–2178 (2016). https://doi.org/10.1007/s00464-015-4517-y.
18. M. Liu and M. Curet, A review of training research and virtual reality simulators for the da Vinci surgical system, *Teach. Learn. Med.* **27**(1), 12–26 (2015).
19. R. Smith, M. Truong *et al.*, Comparative analysis of the functionality of simulators of the da Vinci surgical robot, *Surg. Endosc.* **29**(4), 972–983 (2015).
20. E. Coste-Maniere, L. Adhami *et al.*, Planning, simulation, and augmented reality for robotic cardiac procedures: The STARS system of the ChIR team, *Semin. Thorac. Cardiovasc. Surg.* **14**(2), 141–156 (2003).
21. S. Suzuki, N. Suzuki *et al.*, Tele-surgery simulation with a patient organ model for robotic surgery training, *The Int. J. Med. Robot. Comput. Assist. Surg. (MRCAS)* **1**(4), 80–88 (2005).
22. W. I. Willaert, R. Aggarwal *et al.*, Simulated procedure rehearsal is more effective than a preoperative generic warm-up for endovascular procedures, *Ann. Surg.* **255**(6), 1184–1189 (2012).
23. J. Lenihan and M. Brower, Web-connected surgery: Using the internet for teaching and proctoring of live robotic surgeries, *J. Robot. Surg.* **6**(1), 47–52 (2011).
24. D. H. Shin, L. Dalag *et al.*, A novel interface for the telementoring of robotic surgery, *BJU Int.* **116**(2), 302–308 (2015).
25. A. M. Jarc, S. H. Shah *et al.*, Beyond 2D telestration: An evaluation of novel proctoring tools for robot-assisted minimally invasive surgery. *Journal of Robotic Surgery*, **10**(2), 103–109 (2016). doi: 10.1007/s11701-016-0564-1.
26. A. Hughes-Hallett, E. K. Mayer *et al.*, The current and future use of imaging in urological robotic surgery: A survey of the European Association of Robotic Urological Surgeons, *Int. J. Med. Robot. Comput. Assist. Surg.* **11**(1), 8–14 (2015).
27. C. R. Mitchell and S. D. Herrell, Image-guided surgery and emerging molecular imaging: advances to complement minimally invasive surgery, *Urol. Clin. North America* **41**(4), 567–580 (2014).
28. M. Beccani, H. Tunc *et al.*, Systematic design of medical capsule robots, *Design and Test, IEEE* **32**(5), 98–108 (2015).
29. F. Qiu, S. Fujita *et al.*, Magnetic helical microswimmers functionalized with lipoplexes for targeted gene delivery, *Adv. Funct. Mater.* **25**(11), 1666–1671 (2015).
30. C. Yiallouras, K. Ioannides *et al.*, Three-axis MR-conditional robot for high-intensity focused ultrasound for treating prostate diseases transrectally, *J. Therapeut. Ultrasound* **3**(1), 1–10 (2015).
31. M. Azizian, M. Khoshnam *et al.*, Visual servoing in medical robotics: A survey. Part I: Endoscopic direct vision imaging — Techniques and applications, *Int. J. Med. Robot. Comput. Assist. Surg.* **10**(3), 263–274 (2014).

The Encyclopedia of Medical Robotics Downloaded from www.worldscientific.com
by 5.61.122.207 on 03/25/22. Re-use and distribution is strictly not permitted, except for Open Access articles.
