# Supplementary material for: Indocyanine green colonic perfusion demonstration following robotic da Vinci X inferior mesenteric artery ligation for the treatment of type II endoleak
Source: Int J Med Robot. 2022 Apr 23;18(4):e2407. doi: 10.1002/rcs.2407 (PMC9541556; doi:10.1002/rcs.2407)
Supplement: Supplementary file 2 — Supporting Information S2 [file RCS-18-e2407-s001.pdf]

Fluorescence imaging for da Vinci X® and da Vinci Xi® surgical systems

Take advantage of real-time visual assessment of vessels, blood flow, and related tissue perfusion

INTUITIVE™

OR reference guide

Table of contents

(Click on link below to jump to a section)

Firefly® technology: Overview

Firefly technology: Activation and adjustment

ICG technical FAQs

ICG administration

Firefly technology indication for use

Clinical application reference and techniques

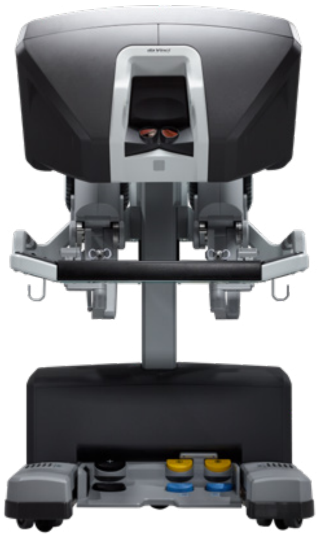

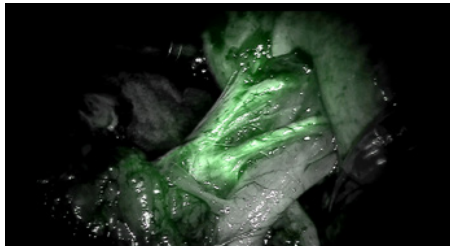

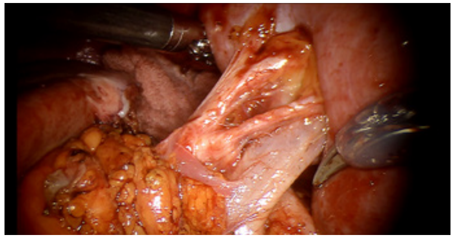

INTUITIVE™

Firefly® technology: Overview

Near-infrared fluorescence guidance

Da Vinci X® and da Vinci Xi® surgical systems with integrated fluorescence imaging capability provide you with real-time endoscopic visible and near-infrared fluorescence imaging. This fluorescence imaging capability provides you with the opportunity for visual assessment of vessels, blood flow, and related tissue perfusion, and at least one of the major extra-hepatic bile ducts. Fluorescence imaging helps enable you to visually assess anatomy.

Firefly® technology: Overview

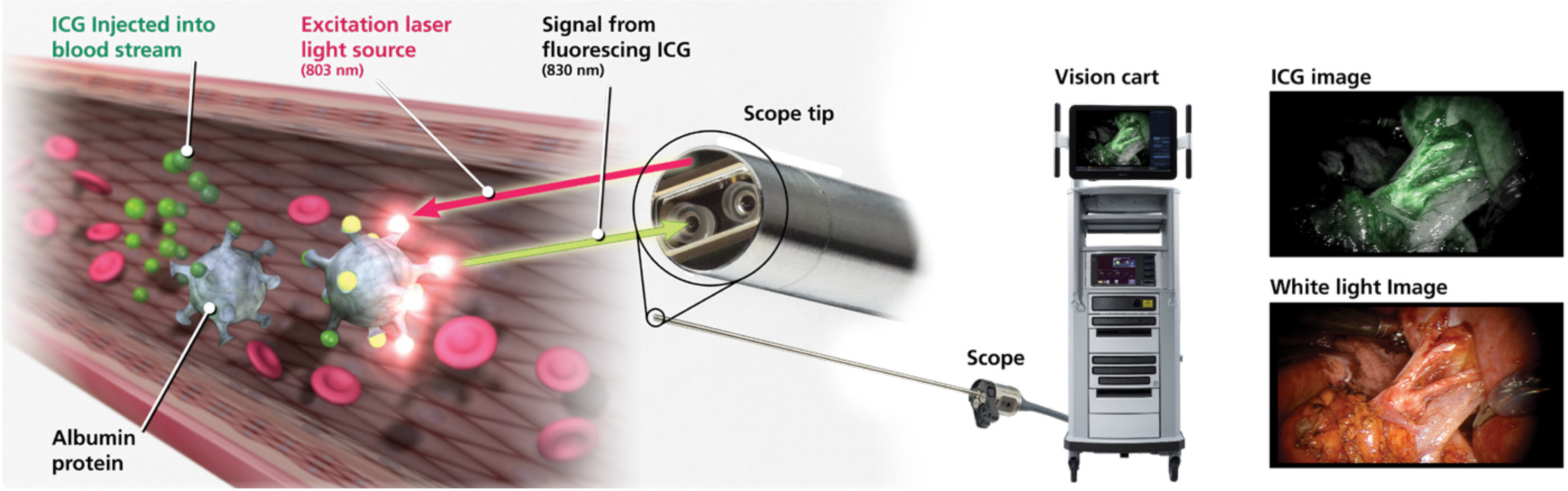

1 Injectable fluorescence dye Indocyanine Green (ICG)

Administered by anesthesiologist through peripheral IV line

Binds to plasma proteins in blood

Emits an infrared signal when excited by laser light in situ

2 Fluorescence-enabled hardware for the da Vinci® X and Xi systems

Illuminator LED with an infrared excitation laser

Da Vinci Xi endoscopes (0° and 30°) come standard with Firefly capability

3 Fluorescence imaging mode on the da Vinci® X and Xi systems

Fluorescing ICG is detected by the da Vinci computer core

Software algorithms colorize the fluorescence signal

Easy switching between normal white light and fluorescence imaging modes from the surgeon console

Firefly® technology: Activation with a da Vinci X® or da Vinci Xi® system

Patient cart

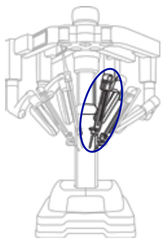

Fluorescence Imaging Equipment

Note
Fluorescence imaging requires a fluorescence-capable Illuminator, camera head and endoscope set up properly. All of this equipment comes standard on X and Xi systems.

Touchscreen view

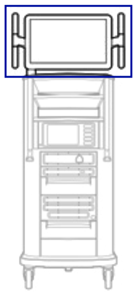

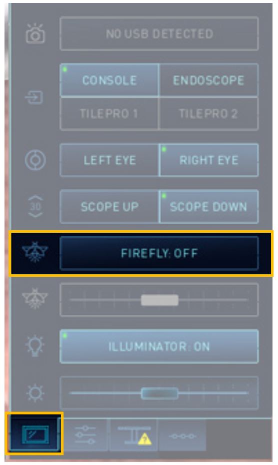

Activating Firefly at Vision Cart

1. Touch the **Display** tab on the Vision cart touchscreen
2. Touch the **Firefly:Off/On** button. If Firefly is already activated, then the button will deactivate Firefly.

Firefly® technology: Activation and adjustment with a da Vinci X® or da Vinci Xi® system

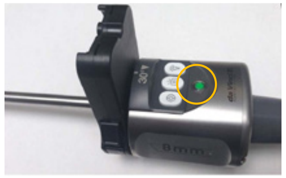

Endoscope LED
The Endoscope LED indicates connection to the Endoscope Controller

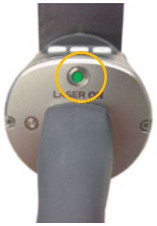

Laser ON LED
When Firefly imaging is active, the “LASER ON” LED indicator on the back of the endoscope illuminates a solid green color.

This indicates that the endoscope tip is emitting laser light. The illumination from the tip of the endoscope visually appears blue in color.

Warning
Avoid looking at light emitted directly from the endoscope or the light guide, which could cause eye injury.

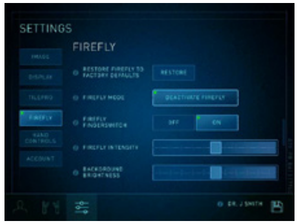

Da Vinci X and Xi endoscopes automatically calibrate.

Da Vinci X and Xi endoscopes (0 degrees and 30 degrees) come standard with Firefly capability.

Ability to adjust Firefly intensity and background brightness.

Firefly® technology: Activation

Surgeon console – touchpad

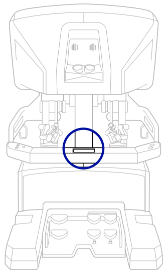

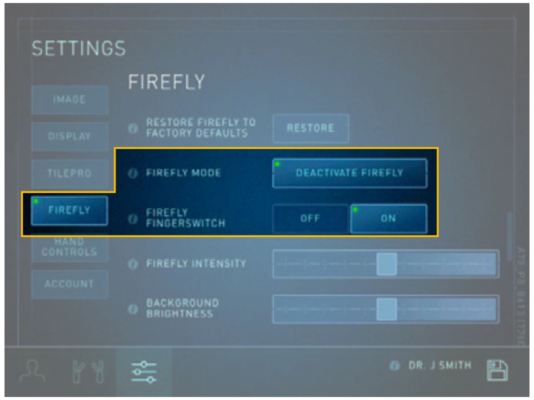

Activating flourescence imaging

1. Select Settings tab
2. Select Firefly button
3. Select Activate button

This toggles the visualization mode between White Light (Off) and Fluorescence (On).

Surgeon console

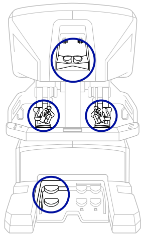

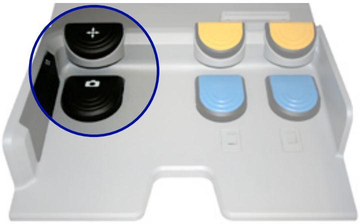

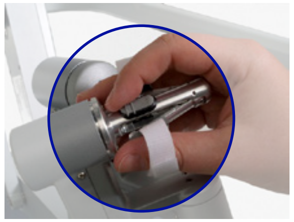

Activating flourescence imaging

1. Press and hold the Camera Control pedal
2. Slide the Finger Switch on the master controls

   Note: Fluorescence Finger Switch must be active on the Surgeon Console

ICG technical FAQs

What is ICG?
Indocyanine green (ICG) is a water-soluble, tricarbocyanine dye with a peak spectral absorption of 800 nm. It has been used clinically since 1959. The chemical name for ICG is 1 H -Benz[e]indolium, 2-[7- [1, 3 -dihydro -1, 1 -dimethyl-3- (4 -sulfobutyl)-2 H -benz[e]indol -2-ylidene]-1,3,5-heptatrienyl]-1,1-dimethyl-3(4-sulfobutyl)-hydroxide, inner salt, sodium salt. ICG has a pH of approximately 6.5 when reconstituted.

Active	Lyophilized green powder containing 25 mg of indocyanine green, aqueous solvent consisting of sterile water for injection
Preservative	None
Inactive	Contains no more than 5.0% sodium iodide

How is it packaged?
ICG is a sterile, lyophilized green powder containing 25 mg of indocyanine green with no more than 5% sodium iodide. It is packaged with aqueous solvent consisting of sterile water for injection used to dissolve the indocyanine green.

Note

For detailed information, refer to manufacturer’s instructions for use.

How is it prepared?
Reconstitute 1 vial of ICG using the aqueous solvent located in the ICG box. The aqueous solvent is sterile water with a pH 5.5 - 6.5 used to dissolve the ICG.

What is the indication for use?
The fluorescence-capable da Vinci Si HD vision system is intended to provide real-time endoscopic-visible and near-infrared fluorescence imaging. This enables surgeons to perform minimally invasive surgery using standard endoscopic-visible light as well as visual assessment of vessels, blood flow and related tissue perfusion using near-infrared imaging.

Are there any allergy or interaction contraindications or warnings?
Contraindications: ICG contains sodium iodide and should be used with caution in patients who have a history of allergy to iodides.

Warnings: Anaphylactic deaths have been reported following ICG administration during cardiac catheterization.

Drug Interactions: Heparin preparations containing sodium bisulfite reduce the absorption peak of ICG in blood and, therefore, should not be used as an anticoagulant when collecting samples for analysis.

$C_{43}H_{47}N_2NaO_6S_2$ Molecular Weight 774.96

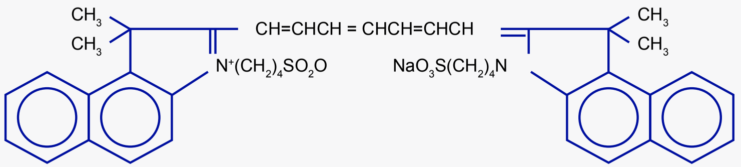

How is ICG administered?
ICG is administered intravascularly via central or peripheral line.

How long will the drug stay in the circulatory system (i.e. half-life)?
ICG has a half-life of 2-5 minutes when bound to blood plasma.

IV injection →	Blood Vessels →	Kidney →	Liver →	Bile →
See within:	5-50 seconds	< 1 min	< 2 min	Tens of minutes
Visibility lasts:	seconds	~ 20 min	1 – 2 hours	1 – 2 hours

What is the typical dose administered?
After reconstitution, a 10 mL vial of ICG contains 2.5 mg per mL of solution. The normal dosing range for fluorescence imaging is 1.0 mL -1.5 mL per intravascular injection of ICG. So a 1.0 mL injection would contain a 2.5 mg dose of ICG.

What is the maximum dosing recommendation?
The maximum dose that can be delivered is 2 mg/Kg of body weight.

Note

Estimation based on animal labs conducted at Intuitive Surgical.
Actual results may vary.

After ICG is prepared, how long will it last?
ICG must be used within 6 hours of reconstitution.

ICG administration

I. Critical product information

It is critical that proper preparation and clear communication occur between the surgeon and the anesthesiologist regarding the administration of the fluorescence imaging agent.

In the Fluorescence Imaging Addendum, PN 550563, familiarize yourself with the information in section **4.3 Warnings/Adverse Reactions Warnings**.

Fluorescence Imaging Agent – Indocyanine Green (ICG)

ICG contains sodium iodide and should be used with caution in patients who have a history of allergy to iodides or iodinated contrast agents

Important Note
ICG should be used with caution in patients with a known allergy or sensitivity to iodides or iodinated contrast agents. Hospital protocols regarding pretreatment for known allergy may apply.

ICG injection may cause a brief fluctuation of digital oxygen saturation immediately after administration

ICG has a half-life of 2 - 5 minutes when bound to plasma proteins

ICG should be used within 6 hours of reconstitution

ICG administration

II. Preparation and dosing of ICG

Preparation prior to the 1st injection

1. Reconstitute ICG with the 10 ml aqueous solution to obtain a 2.5 mg/ml concentration.
2. Withdraw the desired dosage of ICG solution for each planned imaging sequence into separate 3 ml syringes.
3. Withdraw 10 - 12 ml of normal saline for each planned imaging sequence into separate 12 ml syringes.

ICG dosing

Maximum daily dose not to exceed 2 mg/kg per body weight

Typical doses for IV injection could range from 0.5-1.5 ml at 2.5 mg/ml concentration, depending on procedure and patient anatomy (Communicate with surgeon regarding desired dosage)

ICG (Indocyanine Green)

Fluorescence Imaging Components Needed Per Procedure

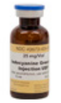
Indocyanine Green (ICG)
25 mg vial

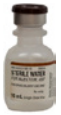
Sterile Water
10 ml vial

Recommended accessories

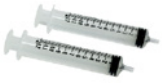
Two 12.0 ml Syringes
w/ Luer Tips

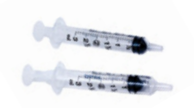
Two 6.0 ml Syringes
w/ Luer Tips

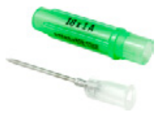
One 18 Gauge
1˝ Needle

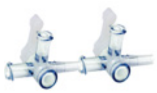
One 2 Gang 4-Way
Stopcock w/ Luer Lock

ICG administration

III. Recommended method of administration

Important note
For optimum fluorescence imaging, each dose of ICG should be injected in a rapid bolus.

Inject ICG either through a central line or a peripheral IV

If you use a peripheral IV, inject through a port close to the IV cannula to ensure rapid infusion

Recommended method of administration for peripheral IV injections

Step 1:
Connect two 3-way stopcocks end to end as close as possible to the IV cannula

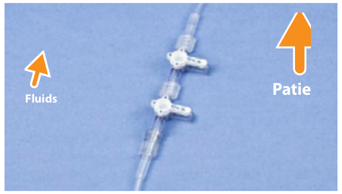

Step 2:
Connect the ICG injection syringe to the stopcock closest to the IV cannula and connect the 12 ml saline flush to the rear stopcock

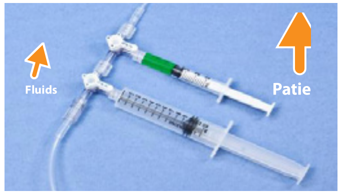

Step 3:
When the surgeon calls for the injection, open the front stopcock and deliver the desired amount of ICG into the line (ensure this does not yet enter the bloodstream by having the saline flush stopcock turned to off on the incoming IV fluid line)

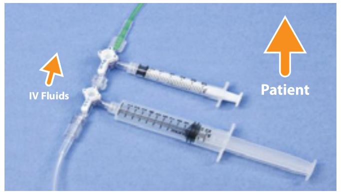

Step 4:
After the ICG injection is delivered, close the stopcock and immediately inject the saline flush to deliver the ICG as a rapid bolus into the bloodstream

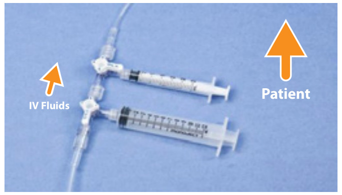

Firefly® indication for use

The da Vinci® Firefly® Imaging System is intended to provide real-time endoscopic visible and near-infrared fluorescence imaging. The da Vinci Firefly Imaging System enables surgeons to perform minimally invasive surgery using standard endoscopic visible light as well as visual assessment of vessels, blood flow, and related tissue perfusion, and at least one of the major extra-hepatic bile ducts (cystic duct, common bile duct and common hepatic duct), using near infrared imaging.

Fluorescence imaging of biliary ducts with the da Vinci Firefly Imaging System is intended for use with standard of care white light and when indicated, intraoperative cholangiography. The device is not intended for standalone use for biliary duct visualization.

Firefly® clinical application reference and techniques

Partial nephrectomy:
Assessment of parenchyma

Partial nephrectomy:
Selective clamping

Partial nephrectomy:
Vessel identification

Sacrocolpopexy:
Vessel identification

Endometriosis resection:
Assessment of peritoneum

Colectomy:
Bowel perfusion assessment

Colectomy:
Vessel identification

Cholecystectomy:
Extrahepatic biliary duct identification

Cholecystectomy:
Cystic artery identification

Tissue perfusion assessment

Thoracic surgery:
Soft tissue perfusion assessment

Lung segmentectomy:
Anatomical segment visualization

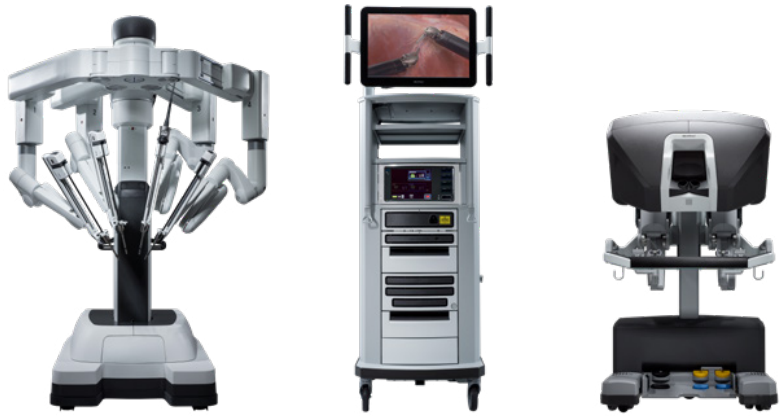

Partial nephrectomy: Parenchymal perfusion assessment

Clinical utility
Using Firefly® technology to assess the healthy parenchyma during tumor excision

Technique & dose
After kidney is de-fatted and area of excision is exposed, inject a test dose of 0.25-0.5 mL depending on body size

Increase dose incrementally until optimal dose for parenchyma assessment is found

Continue with preparation and give this dose again before clamping

Clamp kidney as soon as ICG reaches kidney (don't wait any longer to clamp as ICG will stay indefinitely while clamped, but otherwise washes out)

Time to see
30-50 seconds after peripheral IV injection

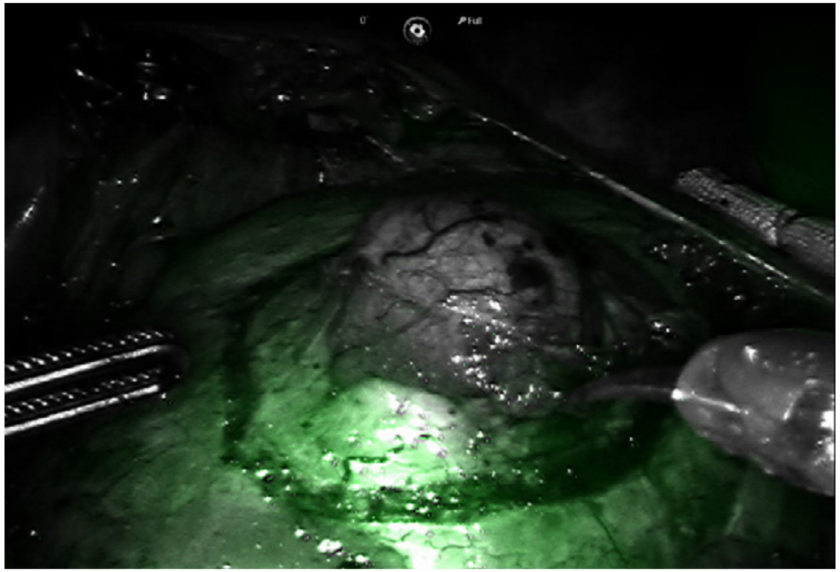

Volume of ICG Injected	Dose of ICG administered	Time to See Once Injected	Length of Time Tissue Flouresces
0.25-0.5 mL(cc)<br>(increase dose as needed)	0.5-1.25 mg	30-50 seconds	• 20 minutes without hilar clamping<br>• Indefinitely when hilum clamped

Note: Maximum dose patient can receive is 2 mg per kilogram of body weight.

Partial nephrectomy: Selective arterial clamping

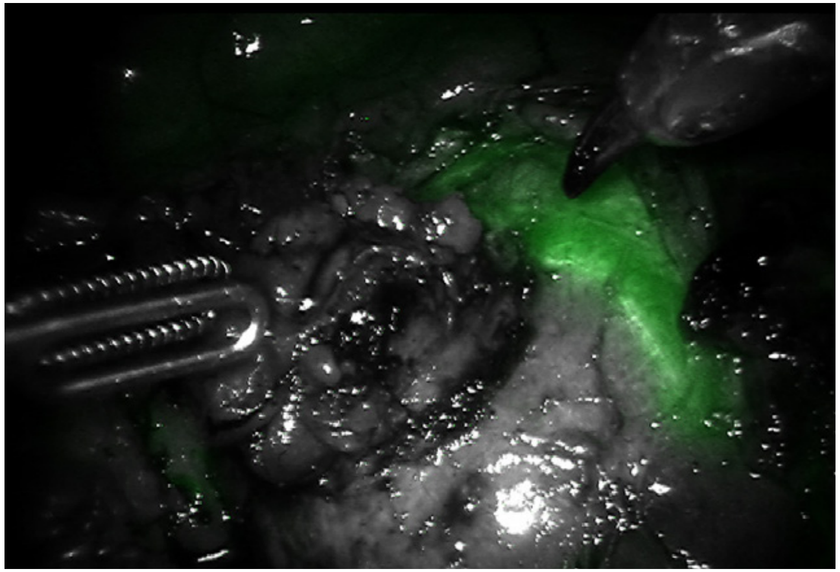

Clinical utility

Using Firefly® technology to identify areas of perfusion from areas of occlusion as means to localize warm ischemia to specific regions of the kidney

Technique & dose

Extend hilar dissection lateral to expose individual branching artery

Surgeon identifies the arterial branch perfusion region of kidney where tumor resides

Surgeon clamps arterial branch then administers a 1.5 mL (3.75 mg) dose of ICG

Firefly technology is used to confirm adequate occlusion of kidney region where tumor resides

If region is not occluded, unclamp and clamp the main branch of renal artery before excising the tumor

Time to see

30-50 seconds after peripheral IV injection

Volume of ICG injected	Dose of ICG administered	Time to see once injected	Length of time tissue flouresces
1.50-2.0 mL(cc)	3.75-5.0 mg	30-50 seconds	• 20 minutes without hilar clamping<br>• Indefinitely when hilum clamped

Note: Maximum dose patient can receive is 2 mg per kilogram of body weight.

Partial nephrectomy: Vessel identification

Clinical utility
Using Firefly® technology to identify arterial and venous structures of the renal hilum including any aberrant vasculature

Technique & dose
Before, during or after dissection of the renal hilum, inject a 1.5 mL (3.75 mg) dose followed by a 10 mL saline flush

Use Firefly technology to confirm complete dissection and identification of all vascular structures

Time to see
30-50 seconds after peripheral IV injection

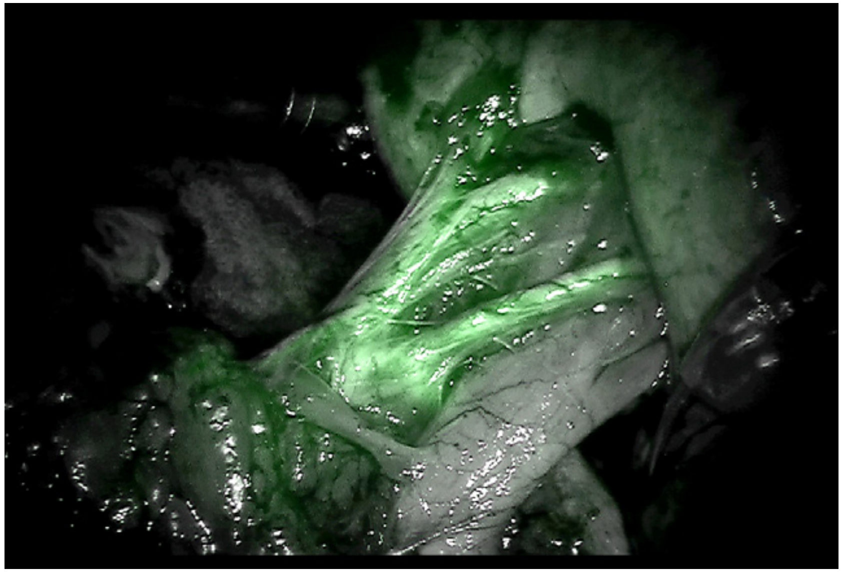

Volume of ICG injected	Dose of ICG administered	Time to see once injected	Length of time tissue flouresces
1.50-2.0 mL(cc)<br>Followed by 10 mL saline flush	3.75-5.0 mg	30-50 seconds	2-5 minutes

Note: Maximum dose patient can receive is 2 mg per kilogram of body weight.

Sacrocolpoplexy: Vessel identification

Clinical utility
Using Firefly® technology to identify the mid-sacral vessels on the sacral promontory to aid in dissection

Technique & dose
Prior to or during preparation of sacral promontory inject a 3 mL (7.5 mg) dose of ICG followed by 10 mL saline flush to identify mid-sacral vessels

Time to see
30-50 seconds after peripheral IV injection

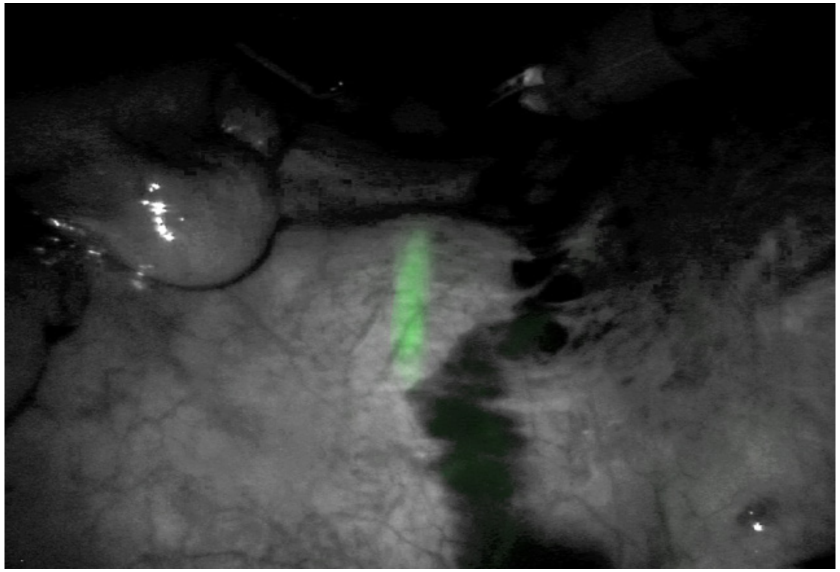

Volume of ICG injected	Dose of ICG administered	Time to see once injected	Length of time tissue flouresces
1.50-2.0 mL(cc)<br>Followed by 10 mL saline flush	3.75-5.0 mg	30-50 seconds	2-5 minutes

Note: Maximum dose patient can receive is 2 mg per kilogram of body weight.

Endometrial resection: Assessment of peritoneum

Clinical utility
Using Firefly® technology to identify areas of hypervascularity on the surface of the peritoneum

Technique & dose
Assess peritoneum in white light mode

In Firefly mode, inject a 1-1.5 mL (2.5-3.75 mg) dose of ICG

Scan the peritoneum for areas of hypervascularity, which are identified by an accumulation of ICG and more intense fluorescence signal

Inject multiple doses as needed

Time to see
30-50 seconds after peripheral IV injection

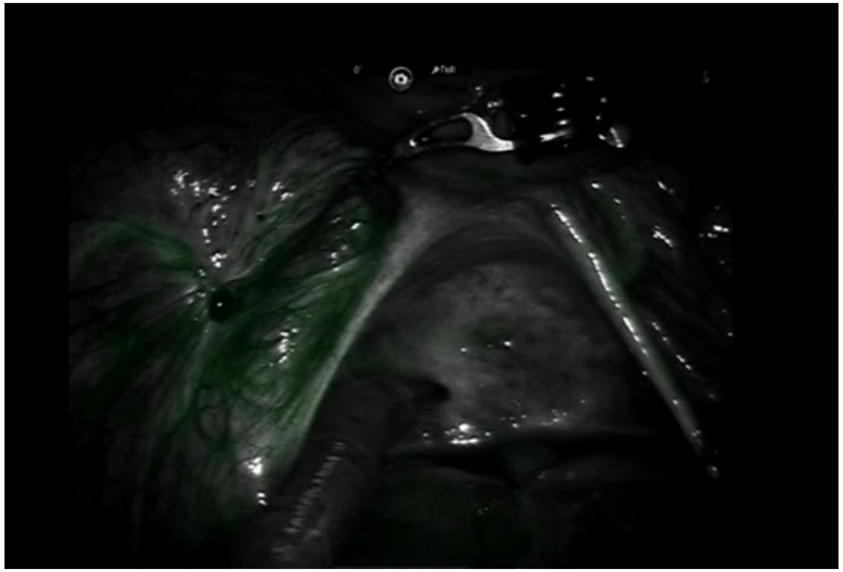

Volume of ICG injected	Dose of ICG administered	Time to see once injected	Length of time tissue flouresces
1.0-1.5 mL(cc)	2.50-3.75 mg	30-50 seconds	2-5 minutes

Note: Maximum dose patient can receive is 2 mg per kilogram of body weight.

Colectomy: Bowel perfusion assessment

Clinical utility
Using Firefly® technology to identify areas of perfusion from areas of ischemia during colectomy procedures

Technique & dose
After mobilization of proximal colon identify point of transection in white light mode

In Firefly mode, inject a 3 mL (7.50 mg) dose of ICG and assess area of perfusion versus areas of de-vascularized (ischemic) colon

Inject multiple times as needed.

Time to see
30-50 seconds after peripheral IV injection

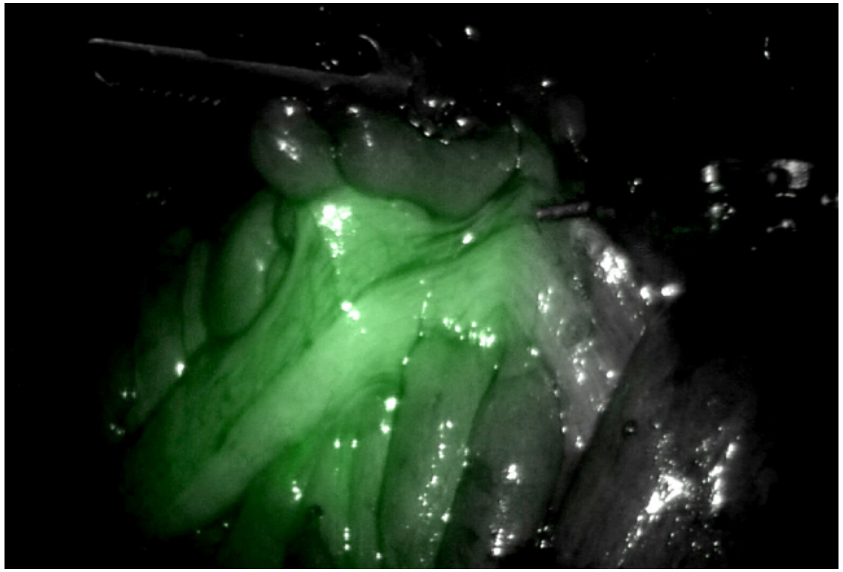

Volume of ICG injected	Dose of ICG administered	Time to see once injected	Length of time tissue flouresces
3.0 mL(cc)	7.5 mg	30-50 seconds	2-5 minutes

Note: Maximum dose patient can receive is 2 mg per kilogram of body weight.

Colectomy: Vessel identification

Clinical utility
Using Firefly® technology to identify the inferior mesenteric artery to aid in dissection of the mesentery

Technique & dose
Prior to or during dissection of the mesentery, inject a 2 mL (5 mg) dose of ICG followed by 10 mL saline flush to identify the IMA

Time to see
30-50 seconds after peripheral IV injection

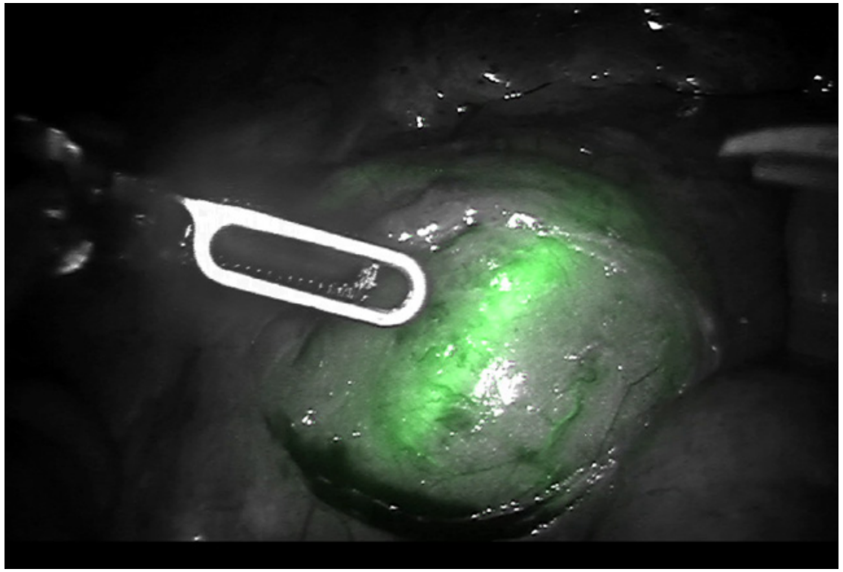

Volume of ICG injected	Dose of ICG administered	Time to see once injected	Length of time tissue flouresces
1.50-2.0 mL(cc)<br>Followed by 10 mL saline flush	3.75-5.0 mg	30-50 seconds	2-5 minutes

Note: Maximum dose patient can receive is 2 mg per kilogram of body weight.

Cholecystectomy: Identification of extrahepatic biliary ducts

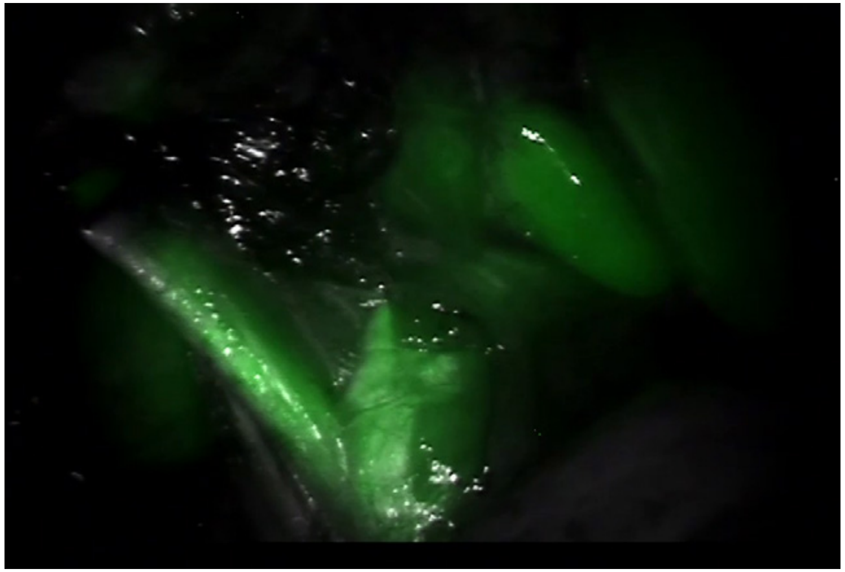

Clinical utility
Using Firefly® technology to identify at least one of the extrahepatic biliary ducts (cystic, common bile and common hepatic duct)

Technique & dose
Systemic injection of 1.5-2.0 mL (3.75-5.0 mg) at least 45 minutes prior to start of the case

Use Firefly technology during dissection of Calot's triangle to identify extrahepatic biliary ducts

Time to see
At least 45 minutes after peripheral IV injection

Volume of ICG injected	Dose of ICG administered	Time to see once injected	Length of time tissue flouresces
1.50-2.0 mL(cc)	3.75-5.0 mg	45 minutes	2-3 hours

Note: Maximum dose patient can receive is 2 mg per kilogram of body weight.

Note: Fluorescence imaging of biliary ducts with the da Vinci Fluorescence Imaging Vision System is intended for use with standard of care white light and, when indicated, intraoperative cholangiography. The device is not intended for standalone use for biliary duct visualization.

Cholecystectomy: Identification of cystic artery

Clinical utility
Using Firefly® technology to identify the cystic artery

Technique & dose
Use Firefly technology during dissection of Calot's triangle to identify the cystic artery

Systemic injection of 1.5 mL (3.75 mg) immediately followed by a 10 mL saline flush

Time to see
Approximately 30-60 seconds after peripheral IV injection

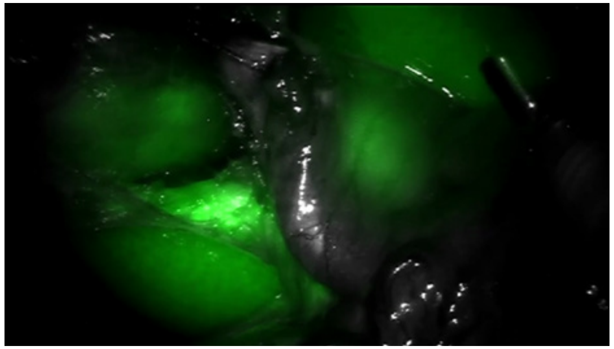

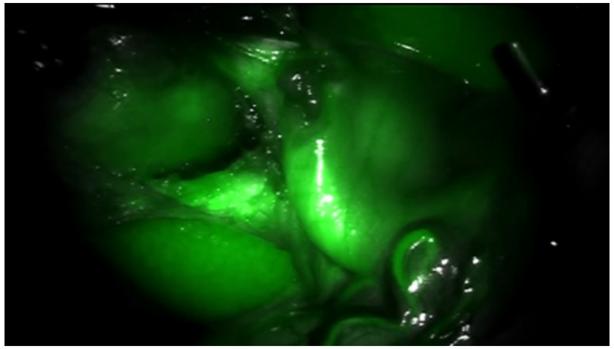

Volume of ICG injected	Dose of ICG administered	Time to see once injected	Length of time tissue flouresces
1.50 mL(cc)<br>Followed by 10 mL saline flush	3.75 mg	30-60 seconds	2-5 minutes

Note: Maximum dose patient can receive is 2 mg per kilogram of body weight.

Inguinal hernia repair: Assessment of tissue perfusion

Clinical utility
Using Firefly® technology to assess vascularity of tissue

Technique & dose
Use Firefly during takedown of avascular structures to identify vascular vs avascular tissues

Systemic injection of 1.5-2 mL immediately followed by a 10 mL saline flush

Time to see
Approximately 30-60 seconds after peripheral IV injection

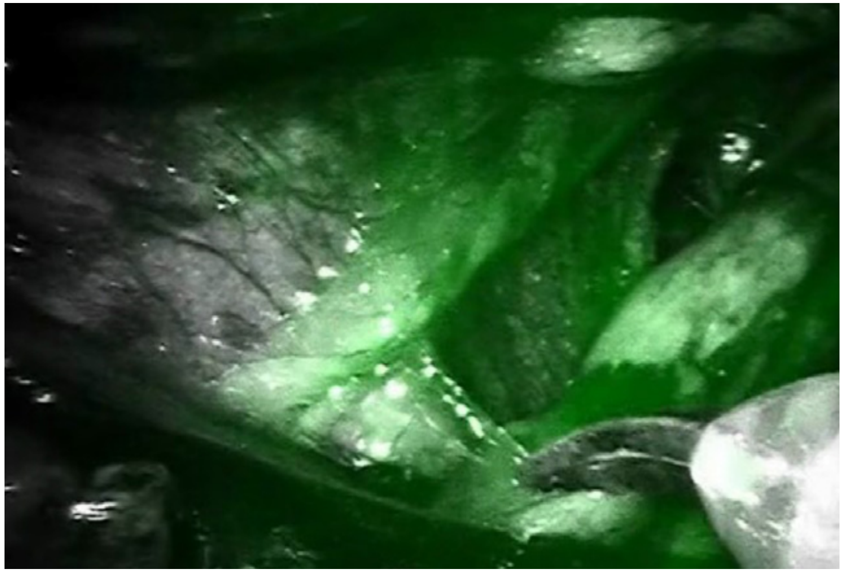

Volume of ICG injected	Dose of ICG administered	Time to see once injected	Length of time tissue flouresces
1.50-2.0 mL(cc)<br>Followed by 10 mL saline flush	3.75-5.0 mg	30-60 seconds	3-5 minutes

Note: Maximum dose patient can receive is 2 mg per kilogram of body weight.

Thoracic surgery: Soft tissue perfusion assessment

Clinical utility
Using Firefly® technology to identify areas of perfusion from areas of ischemia during thoracic surgery

Technique & dose
After preparation of proximal stomach and distal esophagus, inject a 3 mL (7.5 mg) dose of ICG to assess area of perfusion versus areas of de-vascularized (ischemic) tissue

Inject multiple times as needed:
- Injection #1: proximal stomach
- Injection #2: distal esophagus
- Injection #3: during or after anastomosis

Time to see
30-50 seconds after peripheral IV injection

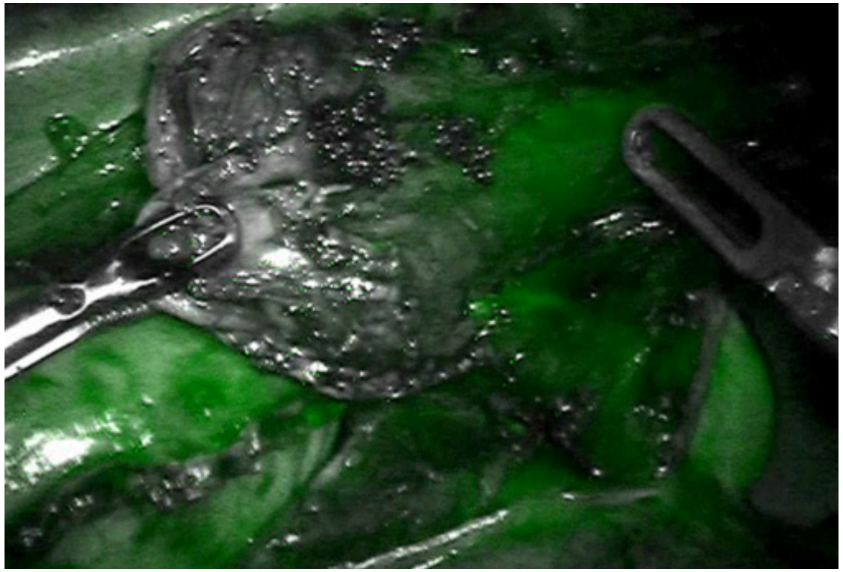

Volume of ICG injected	Dose of ICG administered	Time to see once injected	Length of time tissue flouresces
3.0 mL(cc)	7.5 mg	30-50 seconds	2-5 minutes

Note: Maximum dose patient can receive is 2 mg per kilogram of body weight.

Lung segmentectomy: Anatomical segment visualization

Clinical utility
Using Firefly® technology to identify the anatomical segment

Technique & dose
Use Firefly technology after clamping off or taking down vessel to identify perfused tissue vs. non-perfused tissue

Systemic injection of 5-8 mL immediately followed by a 10 mL saline flush

Time to see
Approximately 30-60 seconds after peripheral IV injection

After approximately 3-5 minutes, ischemic demarcation may be less prominent as ICG diffuses throughout lung tissue

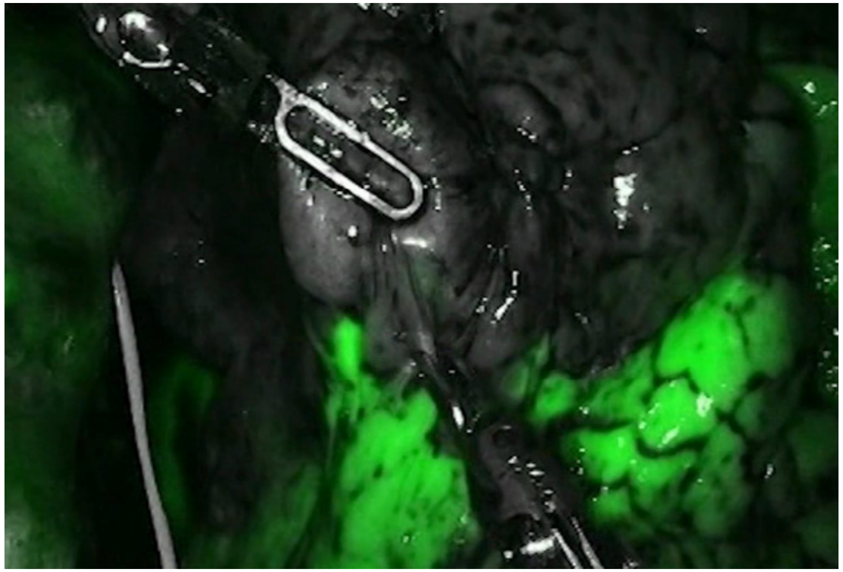

Volume of ICG injected	Dose of ICG administered	Time to see once injected	Length of time tissue flouresces
5-8 mL(cc)<br>Followed by 10 mL saline flush	12.5-20 mg	30-60 seconds	3-5 minutes

Note: Maximum dose patient can receive is 2 mg per kilogram of body weight.

Surgical risks

Nephrectomy: renal insufficiency, urine leak, splenic, hepatic or pancreatic laceration, bowel injury, pneumothorax, diaphragmatic injury, urinary fistula, urinoma, renal infarction, lymphocele

Sacrocolpopexy: mesh erosion/infection (if mesh used in repair) with need for re-operation, rectal injury, bladder injury, rectocele, cystocele, urinary tract injury, vaginal cuff dehiscence, urinary incontinence, hematoma (retropubic, perineal or other).

Endometriosis resection: bowel injury, bladder injury, urinary tract injury

Bowel Resection and Other Colorectal Procedures (Colectomy, Sigmoidectomy, Low Anterior Resection, APR, Intersphincteric Resection, Proctectomy, Rectopexy): anastomotic leak, anastomotic stricture, colorectal or anorectal dysfunction

Cholecystectomy: common bile duct injury; bile leak; pancreatitis, retained common bile duct stones

Hernia Repair (ventral, incisional, umbilical, inguinal): recurrence, bowel injury, mesh infection, urinary retention. For inguinal hernia repair: testicular injury

Pulmonary Resection (Wedge Resection, Segmentectomy, Lobectomy): persistent air leak, pneumonia, prolonged mechanical ventilation >48 hours, atrial fibrillation, acute respiratory distress syndrome (ARDS), chylothorax, re-intubation, arrhythmias, bronchopleural fistula, phrenic nerve injury, esophageal injury, difficulty breathing, collapsed lung, pulmonary volvulus, recurrent laryngeal nerve injury leading to vocal cord dysfunction

Important safety information

Serious complications may occur in any surgery, including da Vinci Surgery, up to and including death. Examples of serious or life-threatening complications, which may require prolonged and/or unexpected hospitalization and/or reoperation, include but are not limited to, one or more of the following: injury to tissues/organs, bleeding, infection and internal scarring that can cause long-lasting dysfunction/pain.

Risks specific to minimally invasive surgery, including da Vinci® Surgery, include but are not limited to, one or more of the following: temporary pain/nerve injury associated with positioning; a longer operative time, the need to convert to an open approach, or the need for additional or larger incision sites. Converting the procedure could result in a longer operative time, a longer time under anesthesia, and could lead to increased complications. Contraindications applicable to the use of conventional endoscopic instruments also apply to the use of all da Vinci instruments.

For Important Safety Information, indications for use, risks, full cautions and warnings, please also refer to www.davincisurgery.com/safety and www.intuitivesurgical.com/safety.

Individual surgical results may vary.

da Vinci Xi® system precaution statement

The demonstration of safety and effectiveness for the specific procedure(s) discussed in this material was based on evaluation of the device as a surgical tool and did not include evaluation of outcomes related to the treatment of cancer (overall survival, disease-free survival, local recurrence) or treatment of the patient's underlying disease/condition. Device usage in all surgical procedures should be guided by the clinical judgment of an adequately trained surgeon.

Firefly® fluorescence imaging

The da Vinci® Fluorescence Imaging Vision System (Firefly® Fluorescence Imaging) is intended to provide real-time endoscopic visible and near-infrared fluorescence imaging. The da Vinci Fluorescence Imaging Vision System enables surgeons to perform minimally invasive surgery using standard endoscopic visible light as well as visual assessment of vessels, blood flow, and related tissue perfusion, and at least one of the major extra-hepatic bile ducts (cystic duct, common bile duct and common hepatic duct), using near infrared imaging.

Fluorescence imaging of biliary ducts with the da Vinci Fluorescence Imaging Vision System is intended for adjunctive use only, in conjunction with standard of care white light and when indicated, with intraoperative cholangiography. The device is not intended for standalone use for biliary duct visualization.

Intuitive's ICG packs are available for sale in the U.S. ONLY. Intuitive's ICG packs are cleared for commercial distribution in the U.S. for use in combination with the fluorescence-capable da Vinci HD vision system and Firefly integrated hardware. Intuitive-distributed ICG contains necessary directions for use of ICG with Firefly Fluorescence Imaging. Using generic ICG with Firefly Fluorescence Imaging is considered off-label and is not recommended. Anaphylactic deaths have been reported following ICG injection during cardiac catheterization. Total ICG dosage should not exceed 2 mg/kg per patient. Anaphylactic or urticarial reactions have been reported in patients with or without histories of allergy to iodides.

© 2018 Intuitive Surgical, Inc. All rights reserved. Product names are trademarks or registered trademarks of their respective holders.
